# Transgressive segregation, hopeful monsters, and phenotypic selection drove rapid genetic gains and breakthroughs in predictive breeding for quantitative resistance to *Macrophomina* in strawberry

**DOI:** 10.1093/hr/uhad289

**Published:** 2024-01-03

**Authors:** Steven J Knapp, Glenn S Cole, Dominique D A Pincot, Christine Jade Dilla-Ermita, Marta Bjornson, Randi A Famula, Thomas R Gordon, Julia M Harshman, Peter M Henry, Mitchell J Feldmann

**Affiliations:** Department of Plant Sciences, University of California, Davis, One Shields Avenue, Davis, CA 95616, USA; Department of Plant Sciences, University of California, Davis, One Shields Avenue, Davis, CA 95616, USA; Department of Plant Sciences, University of California, Davis, One Shields Avenue, Davis, CA 95616, USA; Department of Plant Sciences, University of California, Davis, One Shields Avenue, Davis, CA 95616, USA; Crop Improvement and Protection Research, USDA-ARS, 1636 E. Alisal Street, CA 93905, USA; Department of Plant Sciences, University of California, Davis, One Shields Avenue, Davis, CA 95616, USA; Department of Plant Sciences, University of California, Davis, One Shields Avenue, Davis, CA 95616, USA; Department of Plant Pathology, University of California, One Shields Avenue, Davis, CA 95616, USA; Department of Plant Sciences, University of California, Davis, One Shields Avenue, Davis, CA 95616, USA; Crop Improvement and Protection Research, USDA-ARS, 1636 E. Alisal Street, CA 93905, USA; Department of Plant Sciences, University of California, Davis, One Shields Avenue, Davis, CA 95616, USA

## Abstract

Two decades have passed since the strawberry (*Fragaria x ananassa*) disease caused by *Macrophomina phaseolina*, a necrotrophic soilborne fungal pathogen, began surfacing in California, Florida, and elsewhere. This disease has since become one of the most common causes of plant death and yield losses in strawberry. The *Macrophomina* problem emerged and expanded in the wake of the global phase-out of soil fumigation with methyl bromide and appears to have been aggravated by an increase in climate change-associated abiotic stresses. Here we show that sources of resistance to this pathogen are rare in gene banks and that the favorable alleles they carry are phenotypically unobvious. The latter were exposed by transgressive segregation and selection in populations phenotyped for resistance to *Macrophomina* under heat and drought stress. The genetic gains were immediate and dramatic. The frequency of highly resistant individuals increased from 1% in selection cycle 0 to 74% in selection cycle 2. Using GWAS and survival analysis, we found that phenotypic selection had increased the frequencies of favorable alleles among 10 loci associated with resistance and that favorable alleles had to be accumulated among four or more of these loci for an individual to acquire resistance. An unexpectedly straightforward solution to the *Macrophomina* disease resistance breeding problem emerged from our studies, which showed that highly resistant cultivars can be developed by genomic selection *per se* or marker-assisted stacking of favorable alleles among a comparatively small number of large-effect loci.

## Introduction

The development of cultivars resistant to diseases caused by necrotrophic fungal pathogens has been challenging in plants because of the lifestyles and harsh infection strategies of the pathogens and characteristically complex and quantitative defense mechanisms of the hosts [1–11]. Studies in several agriculturally important plants have shown that sources of resistance to diseases caused by necrotrophs are uncommon and inherently weak, that genetic variation for resistance tends to be quantitative and limited, and that resistance phenotypes tend to be ambiguous and marginally heritable [2, 6, 12–17]. With these generalizations as a starting point, we initiated studies in 2015 to gain an understanding of the genetic basis of resistance to the disease of strawberry (*Fragaria x ananassa*) caused by the necrotrophic fungal pathogen *Macrophomina phaseolina* [18–21]. This widespread generalist pathogen causes plant death and yield losses in strawberry and numerous other agriculturally important plants, including soybean, groundnut, sunflower, and sorghum [13, 14, 17, 22, 23]. Although universally identified as a necrotroph, the occurrence of a latent life cycle phase suggests that *M. phaseolina* might be hemibiotrophic [23–25]. This nuance aside, the virulent stage is necrotrophic [23]. Our working hypothesis, built on findings in other plants, was that resistance to this pathogen was weak, quantitative, and genetically complex in strawberry.

*Macrophomina* was identified as a serious but geographically limited threat to production in strawberry in the decade before the global phase-out of methyl bromide fumigation began in 2005 [26–28]. This ozone-depleting substance had been widely used since 1960 as a soil fumigant in strawberry and other agriculturally important plants to limit losses to diseases caused by soilborne pathogens [29–33]. *Macrophomina* was virtually unknown as a strawberry disease problem in California, Florida, and Spain until initial reports began surfacing in 2004 [20, 34–36], but had been reported earlier in Israel, Egypt, and elsewhere [27, 28]. This disease has since become one of the most serious and widespread causes of plant death and yield losses in strawberry, particularly in warm climates [18, 19, 21, 28, 34, 36–39]. The escalating importance of *Macrophomina*-caused diseases in agriculturally important plants appears to be strongly correlated with an increase in abiotic stresses aggravated by climate change, especially heat and drought stress [22, 23, 28, 40–44].

The rapid emergence of *Macrophomina* as a disease problem in strawberry has meant that the search for breeding solutions, the identification and utilization of sources of resistance in breeding, and studies to elucidate the genetics of resistance have been limited [28, 45, 46]. Nevertheless, a significant breakthrough was reported in an earlier genetic study [46]. Nelson et al. [46] identified three large-effect loci (*MP1*, *MP2*, and *MP3*) that were associated with quantitative resistance to *Macrophomina* in strawberry. *MP1* and *MP2* were identified by quantitative trait locus (QTL) mapping in elite $\times$ elite full-sib families, whereas *MP3* was identified by QTL mapping in elite $\times$ exotic full-sib families. The favorable *MP3* allele was predicted to have been transmitted by the exotic parent (FVC 11–58), an interspecific hybrid between *F. virginiana* and *F. chiloensis* ecotypes [47]. Using area under the disease progress curve (AUDPC) estimates, they showed that symptom development could be slowed and that susceptibility could be decreased by stacking favorable *MP1* and *MP2* alleles. Finally, they suggested that resistance to *Macrophomina* might be further increased by stacking favorable alleles among the three loci.

The studies described here were undertaken to accelerate the development of strawberry cultivars resistant to *Macrophomina*, develop deeper insights into the genetics of resistance to *Macrophomina*, and identify genome-informed solutions to the *Macrophomina* disease resistance breeding problem in strawberry [15, 48]. *Macrophomina* has presumably been omnipresent in soils for millennia without causing disease [23], at least not frequently, and without posing a serious risk to strawberry production. However, since gaining a foothold in California and other parts of the world over the last 20 years [20, 27, 35], the development of *Macrophomina* resistant strawberry cultivars has become imperative. Here we report findings from an in-depth survey of genetic variation for resistance to *Macrophomina* and from analyses of phenotypic selection experiments that shed light on the genetics of resistance to *Macrophomina*. Our studies were initiated by screening diverse *F. chiloensis*, *F. virginiana*, and *F.*$\times$*ananassa* genetic resources (asexually propagated hybrid individuals) for resistance to *Macrophomina* under high summer temperatures and induced drought stress. We report significant breakthroughs in breeding for resistance to *Macrophomina* that were facilitated by the development of straightforward and effective phenotyping protocols, the identification of multiple elite and exotic sources of favorable alleles for enhancing resistance, extreme transgressive segregation, the aggregation and accumulation of favorable alleles from multiple sources of resistance into elite genetic backgrounds, rapid genetic gains from phenotypic selection, and the discovery of several loci that appear to have been targeted by phenotypic selection for increased resistance to *Macrophomina*. Our genetic studies were facilitated by using a high-throughput genotyping array populated with single nucleotide polymorphisms (SNPs) physically anchored to a haplotype-phased octoploid reference genome developed for the cultivar ‘Royal Royce’ (https://phytozome-next.jgi.doe.gov/info/FxananassaRoyalRoyce_v1_0) [49]. Lastly, we propose formulaic genome-informed solutions to the *Macrophomina* disease resistance breeding problem in strawberry, in addition to addressing open questions and discussing the limitations of our study.

## Results

### Selection cycle zero: Assessing the frequency and strength of resistance to *Macrophomina* in strawberry

**Figure 1:**
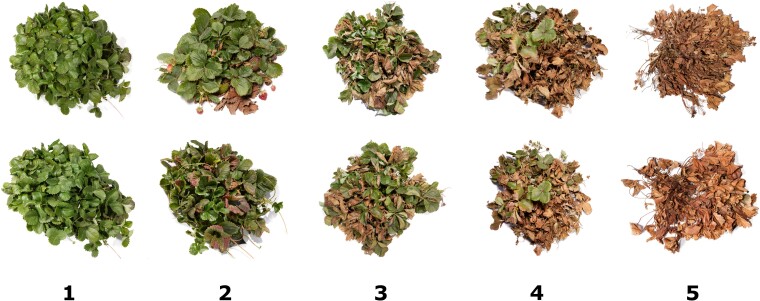
Symptoms of the crown rot disease of strawberry caused by *Macrophomina phaseolina*. The strawberry plants depicted here are selection cycle one progeny observed August 30, 2022 in Salinas, CA. The ordinal scores applied to visual symptoms are shown below the photographic images, where 1 = highly resistant (symptomless) and 5 = highly susceptible (dead).

**Figure 2:**
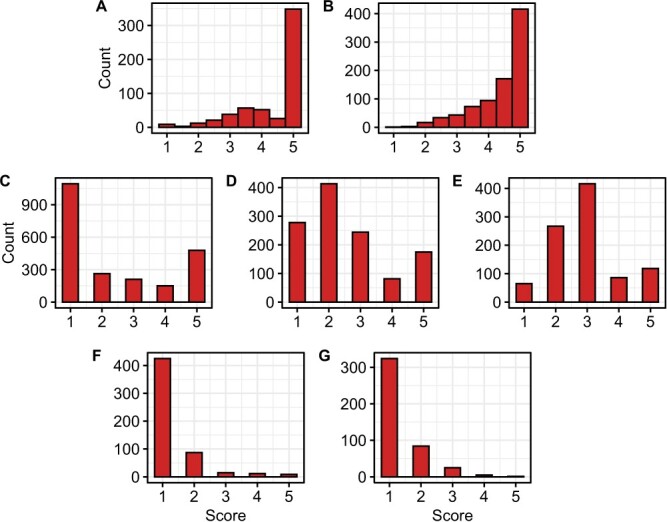
Histograms for ordinal *Macrophomina* resistance scores among selection cycle 0, 1, and 2 individuals. (A-B) The data displayed for C0 populations are phenotypic means estimated from four clonal replicates per individual. (C-G) The data displayed for C1, C2, and C1R populations are phenotypic observations of unreplicated seed-propagated individuals within full-sib families. (A) Histogram for 565 octoploid strawberry germplasm accessions phenotyped in Davis, CA in the summer of 2016. (B) Histogram for 853 octoploid germplasm accessions phenotyped in Irvine, CA in the summer of 2017. (C) Histogram for 2211 individuals within 17 C1 full-sib families phenotyped in Davis, CA in the summer of 2019. (D) Histogram for 1260 individuals within 25 C1R full-sib families phenotyped in Davis, CA in the summer of 2022. (E) Histogram for 960 individuals within 20 C1R full-sib families phenotyped in Salinas, CA in the summer of 2022. (F) Histogram for 560 individual within 18 C2 full-sib families phenotyped in Davis, CA in the summer of 2021. (G) Histogram for 441 individuals within 18 C2 full-sib families phenotyped in Salinas, CA in the summer of 2021.

Our data suggests that resistance to *Macrophomina* is rare among wild relatives, cultivars, and other individuals preserved in clonal octoploid strawberry gene banks (Figs. 1-2; Supplemental Files S1-S2). This conclusion was reached by screening artificially inoculated clonal replicates of a diverse collection of 853 genetic resources (clonally preserved hybrid individuals and ecotypes) for disease symptoms under high summer temperatures and induced drought stress. These selection cycle 0 (C0) individuals were the source of the elite and exotic parents selected for developing full-sib families for the selection cycle one (C1) population (Supplemental File S3). The phenotypes reported herein for the C0, C1, and cycle 2 (C2) populations were observed in the precipitation-free months of June and July in Davis, CA when daily maximum temperatures were in the 27°C to 42°C range (https://www.weather.gov/wrh/climate). We discovered that differences in resistance phenotypes were most effectively expressed and differentiated when moderate drought stress was induced by decreasing irrigation by 50% to 70%, enough to cause mild wilting without leaf scorch in resistant hybrid checks (Fig. 4A).

When our study was initiated in 2015, there was a dearth of information on sources of resistance to *Macrophomina* and an absence of information on the genetics of resistance to this pathogen in strawberry [19, 21, 37, 38, 46]. To rectify this and build the foundation for the present study, we drew upon several sources of information to select the widest possible array of individuals preserved in clonal gene banks at the University of California, Davis and USDA National Plant Germplasm System (https://www.ars.usda.gov/) (Supplemental File S1): genetic relationships estimated from pedigree records (coancestry coefficients) and genome-wide single nucleotide polymorphism (SNP) profiles; breeding program origin for *F.*$\times$*ananassa* germplasm; and phylogenetic and geographic origin for ecotypes of the wild ancestors (*F. chiloensis* and *F. viginiana*) [50, 51]. Of the 853 octoploid clonal genetic resources (C0 individuals) phenotyped in our study, 265 were acquired from the USDA collection, 588 were preserved in the UC Davis collection, 778 were *F.*$\times$*ananassa*, 39 were *F. chiloensis*, and 36 were *F. virginiana* (Supplemental File S1).

**Figure 3:**
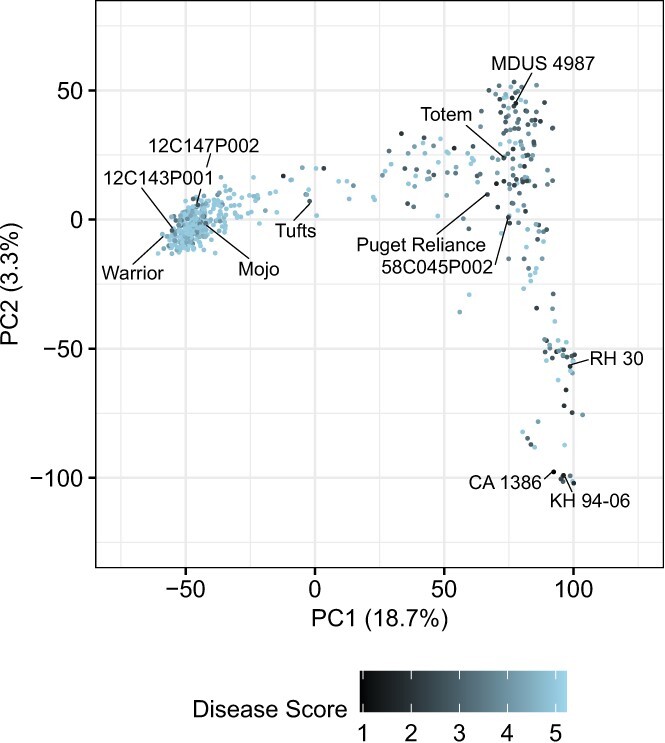
V-shaped distribution of genetic relationships among 584 individuals in the cycle 0 population visualized by principal component analysis of a genomic relationship matrix (GRM) estimated from 48 340 single nucleotide polymorphisms genotyped with a 50 K Axiom array. Score for the first two principal components are shown. The far left upper tip of the V is comprised of modern UC cultivars and hybrids, whereas the far right lower tip of the V is comprised of *Fragaria chiloensis* ecotypes (e.g., CA 1386 and KH 94–06) with *F. virginiana* ecotypes (e.g., RH 30) falling slightly upstream. The vertex of the V is comprised primarily of early UC cultivars and hybrids (e.g., 58C045P002) and early and modern non-UC cultivars (e.g. ‘Totem’). Estimated marginal means (EMMs) for disease score, which ranged from highly resistant (1) to highly susceptible (5), were estimated from four clonal replicates/individual in two disease resistance screening studies (Davis, CA in 2016 and Irvine, CA in 2017). The within and across location EMMs for every individual are tabulated in Supplemental File S1

Using bare-root clones artificially inoculated with the pathogen, 99% of the C0 individuals screened in our studies developed moderate to severe disease symptoms with ordinal disease score means ($\overline{y}$) in the 2.0 to 5.0 range (Fig. 1-2; Supplemental File S1). Eighty percent of these individuals developed severe symptoms ($4.0\le \overline{y}\le 5.0$) and most were killed by the pathogen ($y=5.0$). The resistance score distributions in both screening experiments were left-skewed with disease score medians of 5 and means of 4.4 in both locations (Fig. 2A–B). Symptomless individuals ($y=1$) were extremely rare in the C0 population (Supplemental File S1). Less than one percent of the C0 individuals screened in either location had disease score means in the highly resistant range ($1.00\le \overline{y}\le 1.75$) (Supplemental File S1). The most resistant individuals identified in these gene bank screening studies were diverse and included *F. chiloensis* ecotypes (CA1386 and KH94–6), *F. virginiana* ecotypes (RH30 and LH20–1), and early and modern cultivars developed in Maryland (MDUS4987 and MD683), the Pacific Northwest (‘Totem’ and ‘Tillikum’), and California (58C045P002, 12143P001, ‘Tufts’, and ‘Warrior’) (Fig. 3; Supplemental File S1).

**Table 1:** Selection cycle 0, 1, 1R, and 2 population means ($\overline{y}$) and medians ($\tilde{y}$) among $n$ individuals phenotyped for resistance to *Macrophomina* in different locations and years

Cycle	Year	Location	$n$	$\overline{y}$	$\tilde{y}$
C0	2016	Davis	565	4.38	5
	2017	Irvine	853	4.43	5
		Across	565	4.41	5
C1	2019	Davis	2211	2.39	2
C1R	2022	Davis	1260	2.55	2
		Salinas	960	2.92	3
		Across	2220	2.71	3
C2	2021	Davis	560	1.34	1
		Salinas	441	1.35	1
		Across	1001	1.35	1

We used principal component analysis (PCA) of the genomic relationship matrix (GRM) to visualize genetic relationships among 584 C0 individuals that were genotyped with a 50 K Axiom SNP array [49] and phenotyped for resistance to *Macrophomina* in Davis and Irvine, CA (Fig. 3). This analysis reproduced the classic V-shaped distribution and historical genetic groupings previously reported for elite and exotic strawberry germplasm [51]. Broadly speaking, exoticness increases as you trace a path from the upper tip of the V where elite UC individuals are clustered, to the vertex where early UC, non-UC, and heirloom cultivars are clustered, to the lower tip of the V where *F. virginiana* and *F. chiloensis* ecotypes are clustered. The darkness of the points displayed in the principal score plot (Fig. 3) varies according to the estimated marginal means (EMMs) for *Macrophomina* resistance score, where 1 = highly resistant (dark blue) and 5 = highly susceptible (light blue). The darkest points highlight the most resistant individuals identified in our study (their EMMs are tabulated in Supplemental File S1). Our PCA visualization shows that sources of resistance were found across the domestication spectrum, from ecotypes of the wild ancestors to modern UC cultivars found at opposite tips of the V, e.g., from the *F. chiloensis* ecotype CA 1386 at one extreme to the UC cultivar ‘Warrior’ at the other (Fig. 3). Sources of resistance were more common among early UC and non-UC cultivars found near the vertex of the V, from ‘Tufts’ in the upper arm to ‘Totem’ at the vertex to 58C045P002 in the lower right arm (Fig. 3). Although sources of resistance were more common among early UC and other exotic germplasm resources (from ‘Tufts’ to the vertex to CA 1386 in the V), several sources of resistance were identified among elite UC individuals, e.g., 12C147P002, 12C143P001, and ‘Warrior’ (upper left tip of the V to the left of ‘Tufts’ in Fig. 3). This was significant because those individuals supplied elite UC individuals (recipients) for the introgression of novel favorable alleles from exotic sources (donors).

### Heritability of resistance to *Macrophomina* in diverse strawberry Germplasm

The resistance phenotypes observed in the cycle 0 population were predicted to be highly heritable; however, after inspecting the underlying phenotypic observations, we concluded that our heritability estimates for disease score might have been inflated by the high frequency of susceptible individuals ($4.0\le y\le 5.0$) where replicate to replicate variation was minimal. Using the phenotypic observations for the entire C0 population ($1.0\le y\le 5.0$), REML estimates of broad-sense heritability on a clone-mean basis ($\hat{H}$) were 0.45 in Davis, 0.69 in Irvine, and 0.55 across locations. When observations among individuals in the moderately to highly resistant range (1.0 $\overline{y}\le 3.0$; thin left tails of the C0 phenotypic distributions) were inspected, we discovered that the between-location rank correlation for disease score was negative ($\hat{r}=-0.27;p\le 0.0001$). Conversely, when estimated for the entire C0 population, the between-location rank correlation for disease score was positive ($\hat{r}=0.25$; $p=0.004$). Hence, we were not overly confident in the accuracy of the resistance phenotypes observed among C0 individuals and were skeptical of the strength and reproducibility of the resistance phenotypes of the C0 founders that we selected to develop the C1 population (Fig. 2A–B; Supplemental File S1). To highlight our apprehension and the practical ramifications of this uncertainty, the most resistant individual in Davis (98C153P003; $\overline{y}=1.0$) was killed in Irvine ($\overline{y}=4.9$), and only one (CA 1501; $\overline{y}=1.2$) out of 560 individuals screened in both locations (0.18%) had a resistance score below 2.0 (Supplemental File S1).

**Figure 4:**
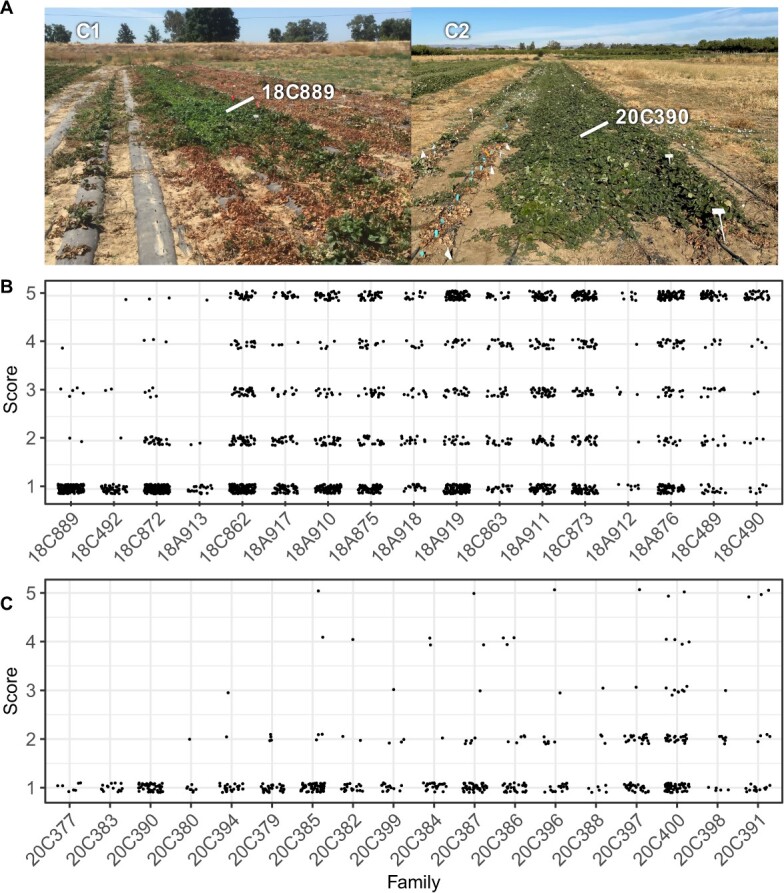
Symptoms and phenotypic distributions for selection cycle 1 and 2 full-sib families screened for resistance to *Macrophomina* in Davis, CA field studies. (A) Selection cycle 1 and 2 full-sib families. The plants shown were observed in July 2018–19 (C1) and 2020–21 (C2). The most resistant full-sib families were 18C889 in the C1 population and 20C390 in the C2 population. (B) Ordinal disease score distributions for 2211 individuals within 17 selection cycle 1 full-sib families observed in 2018–19. (C) Ordinal disease score distributions for 1001 individuals within 18 selection cycle 2 full-sib families observed in 2020–21.

### Selection cycle one: Transgressive segregation, hopeful monsters, and breeding breakthroughs

Although the C0 phenotypic distributions for *Macrophomina* resistance score were continuous (Fig. 2A–B; Table 1), their strongly left-skewed shapes were characteristic of the negative exponential distributions that arise in survival or time-to-event analyses [52, 53]. Such distributions are characteristic of those observed for genetically complex threshold traits [5, 54–56]. We suspected that the strongly left-skewed C0 phenotypic distributions were caused by time-to-event phenotypic variation for resistance, a paucity of favorable alleles, and non-linearity of the ordinal symptom rating scale (Fig. 1). Such non-linearities are common in genetic studies where large-scale visual phenotyping is necessary, large-plot analyses of disease incidence and severity are impractical and unnecessary, and high-throughput phenotyping alternatives are limited, untested, or unproven [5, 57–61]. To increase the probability of selecting parents carrying novel favorable alleles and cope with the uncertain accuracy of C0 resistance phenotypes, we had to relax the truncation selection threshold among prospective selection cycle 1 parents (Supplemental File S1). Sixteen C0 individuals were selected as parents to develop 17 full-sib families for the selection cycle 1 (C1) population (Supplemental File S3). These parents exhibited disease symptoms but were among the least susceptible individuals identified in one or both of our C0 screening studies, e.g. a single clonal replicate of ‘Totem’ was one of a handful of survivors in our Irvine screening study (Fig. 2A–B; Supplemental File S1).

The disease score distribution for the C1 population was approximately bimodal with a disease score median of 2 and mean of 2.4 (Fig. 2C; Table 1), in stark contrast to the left-skewed distributions observed for the C0 population (Fig. 2A-B). The transgressive segregation we observed was dramatic. Symptomless individuals ($y=1$) were observed within every C1 full-sib family (Fig. 4B). Strikingly, 57.0% of the individuals within the four most resistant C1 full-sib families (18C889, 18C492, 18A913, and 18C862) and 91.4% of the individuals within the 18C889 full-sib family were symptomless (Fig. 4B). This suggested that the favorable alleles transmitted by at least one parent were completely dominant ($d/a=1$) or that both parents transmitted additive to completely dominant favorable alleles ($0\le d/a\le 1$), where $d/a$ is the degree of dominance [62]. While novel complementary favorable alleles undoubtedly accounted for a significant fraction of the transgressive phenotypic variation we observed in the C1 population, epistasis and other factors cannot be ruled out [63–65].

The phenotypic variation observed within and among C1 full-sib families (Fig. 4B) and approximately bimodal C1 phenotypic distribution (Fig. 2C) suggested that one or more large-effect loci segregated, a hypothesis explored by using genome-wide association study (GWAS) methods to search for genetic variants in linkage disequilibrium (LD) with causal genes (see below). Lastly, transgressive segregation in the C1 population and genetic gains observed between selection cycles 0 and 1 suggested that our artificial inoculation, field screening, and high-throughput phenotyping protocols identified sources of favorable alleles among diverse gene bank accessions (selection cycle 0 individuals) that were phenotypically unobvious (Supplemental File S1). Such alleles are often hidden in plain sight because of their scarcity and small effects [63, 65, 66], and as we hypothesized, because they are individually necessary but insufficient to confer resistance to this pathogen. The symptomless C1 individuals we selected were ‘hopeful monsters’ [66, 67]: newly created multilocus assemblages of favorable alleles that were aggregated by hybridizing marginally resistant elite and exotic individuals (C0 parents) and exposed by transgressive segregation among their offspring (C1 progeny).

### Selection cycle two: Consolidation, validation, and verification of genetic gains

The validity of the selection cycle 1 breeding breakthrough was substantiated in selection cycle 2 where the frequency of susceptible individuals dropped precipitously (Fig. 2F–G &4C). We developed 18 selection cycle 2 (C2) full-sib families from crosses among 17 symptomless individuals identified in the C1 population (Supplemental File S3). C2 full-sib progeny were screened for resistance to *Macrophomina* in Davis and Salinas, CA using artificial inoculation protocols, screening methods, and study designs identical to those used in selection cycles 0 and 1 (Fig. 2F–G and4C). The phenotypic distributions for the C2 population were strongly right-skewed with disease score medians of 1 (symptomless) in both locations and means of 1.34 in Davis and 1.35 in Salinas (Fig. 2F–G; Table 1).

The decrease in the disease score mean (increase in resistance) between the C0 and C2 populations was highly significant ($4.41-1.35=3.06$; $p\le 0.0001$; Table 1). Of 2203 C2 individuals phenotyped in either location, 1094 were symptomless (49.7%) and 1628 had disease scores $\le 2.0$ (73.9%), a complete reversal of the distributions observed among C0 population individuals (Fig. 2.1A-B & F-G and 2C; Supplemental File S1). Strikingly, 100.0% of the individuals observed in three of the C2 full-sib families (20C377, 20C383, and 20C390) screened in Davis were symptomless ($\overline{y}=1.0$; Fig. 4C). Hence, phenotypic selection under heat and drought stress inverted the highly skewed phenotypic distributions between the C0 founders and C2 descendants and created a population with an exceptionally low frequency of susceptible individuals (Fig. 2–4). The phenotypic changes observed between selection cycles 0 and 2 further suggested that selection may have targeted one or more large-effect loci.

### Selection cycle 1 revisited: Training population winners and losers and a deeper exploration of genetic variation

We developed an independent population of full-sibs (hereafter the C1R population) to sample favorable alleles from a wider sample of prospective donors of novel favorable alleles, replicate and verify the transgressive segregation observed in the C1 population, and build a training population for genomic prediction of *Macrophomina* resistance breeding values. This population was created and analyzed because we suspected that phenotypic selection had profoundly altered allele frequencies and eliminated a significant fraction of the ‘losers’ (susceptible individuals carrying unfavorable alleles) necessary for accurate genomic prediction [68–71]. As an aside, we originally planned to genotype the C1 population (Figs. 2C–4B); however, the DNA samples became a casualty of the COVID pandemic [72–74]. Fortunately, the C1 individuals selected for creating the C1R and C2 populations were preserved and genotyped for the analyses described here.

**Figure 5:**
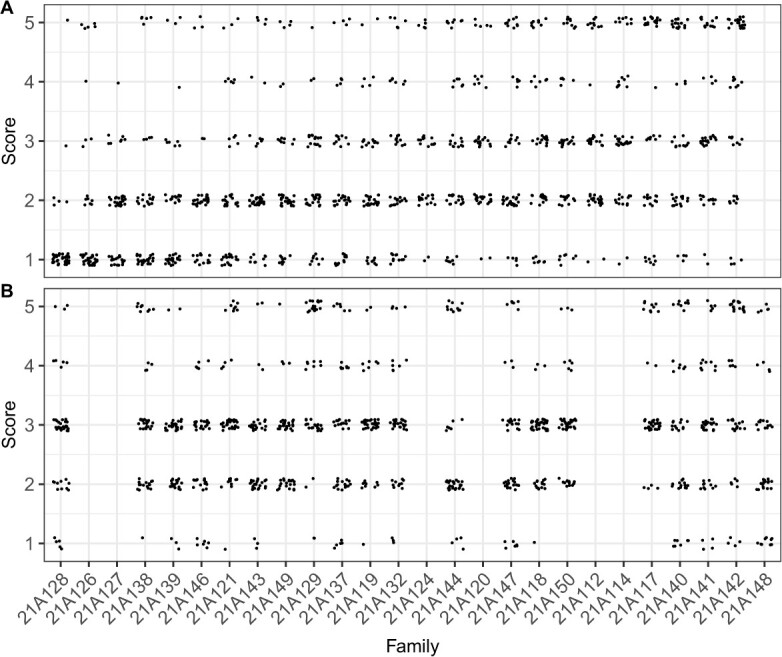
Symptoms observed among replicate selection cycle 1 (C1R) full-sib progeny screened for resistance to *Macrophomina phaseolina* in 2021–22 field studies in Davis and Salinas, CA. (A) Ordinal disease score distributions for 1260 individuals within 25 C1R full-sib families observed July 19, 2022 in Davis, CA. (B) Ordinal disease score distributions for 960 individuals within 20 C1R full-sib families observed October 24, 2022 in Salinas, CA.

An unexpected and challenging aspect of our study was that the C0 population had an overabundance of losers, whereas the C2 population had an overabundance of winners (Fig. 2). Using insights gained from phenotypic selection (Fig. 2 and4) and association genetic analyses of the C0 population (described below), we developed 26 full-sib families among 18 parents that were hypothesized to have a high probability of transmitting novel favorable alleles, transgressively segregating, and producing offspring with normal distributions spanning the entire phenotypic range (Supplemental File S3). That was precisely what we observed among 2220 C1R individuals phenotyped in Davis and Salinas (Fig. 5; Table 1).

The phenotypic distributions for the C1R population were approximately normal with population medians of 2 and means of 2.55 in Davis and 2.46 in Salinas (Fig. 2D-E; Table 1). They differed slightly from the approximately bimodal C1 phenotypic distribution and were more weakly right-skewed than the strongly right-skewed C2 phenotypic distributions (Fig. 2C–G). These findings confirmed our suspicion that C1R full-sib families segregated for complementary favorable alleles among multiple loci affecting resistance to *Macrophomina* (Fig. 2D-E and5). The phenotypic variation observed within and between C1 and C1R full-sib families suggested that favorable alleles for resistance to *Macrophomina* were ‘locked up’ or hidden in diverse elite and exotic genetic resources and exposed by transgressive segregation in the initial hybrid generations. Similar to what we observed among C1 full-sib progeny (Fig. 4), the extreme transgressive segregation observed among C1R full-sib progeny suggested that the selected parents were heterozygous for one or more causal loci and transmitted complementary favorable alleles and that the favorable alleles transmitted by at least one parent were dominant or that both parents transmitted additive or incompletely dominant favorable alleles (Fig. 5).

### Genomic selection as a solution to the *Macrophomina* disease resistance breeding problem

**Figure 6:**
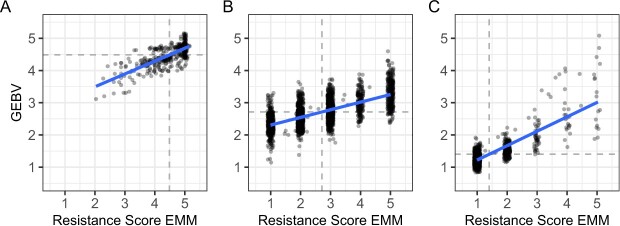
Genomic estimated breeding values (GEBVs) and estimated marginal means (EMMs) for *Macrophomina* resistance score among individuals in the C0 (A), C1R (B), and C2 (C) populations. GEBVs were estimated by genomic best-linear unbiased prediction (G-BLUP). The dashed lines identify the population mean (x-axis) and GEBV mean (y-axis). The solid lines are predicted values from linear regression of GEBV onto resistance score. Statistics are shown for: (A) 565 C0 individuals observed in the 2016 Davis and 2017 Irvine study; (B) 2220 C1R individuals observed in 2022 Davis and Salinas studies; and (C) 1001 C2 individuals observed in 2021 Davis and Salinas studies.

With the effectiveness of phenotypic selection for resistance to *Macrophomina* firmly established (Fig. 4; Table 1), we turned to the problem of assessing the effectiveness of genomic selection, which has the potential to increase throughput and breeding speed (Table 2; Fig. 6) [48]. These analyses were an important element of our studies because selection must be simultaneously applied for resistance to *multiple* diseases caused by soil-borne pathogens [46, 75–79], a process that could be accelerated and strengthened by phenotyping independent samples of training population individuals (e.g., unreplicated seed-propagated progeny) for resistance to different pathogens, and cross-predicting breeding values for different diseases among samples of training population individuals separately phenotyped for individual diseases. The latter is necessary to avoid the confounding effects of symptoms caused by two or more diseases [5, 48]. To that end, we originally developed the C2 full-sib families as a training population to *initiate* long-term genomic selection with continual phenotypic retraining, which we envisioned would be necessary to improve resistance to *Macrophomina*; however, the realized genetic gains from phenotypic selection rendered our original study design unnecessary and steered us towards the formulaic breeding solutions that we explored (Fig. 6; Table 2).

**Table 2:** Genomic selection predictive ability of *Macrophomina* resistance phenotypes estimated by cross-validation among individuals within the C0, C1R, and C2 populations of strawberry. The standard analysis was done by applying genomic-BLUP (G-BLUP) to the genomic relationship matrix (GRM) estimated from a genome-wide sample of 48 340 Axiom array genotyped SNPs. The other G-BLUP analyses were done by using SNPs associated with different subsets of *Macrophomina* resistance loci (*MP1* to *MP10*) to construct foreground (F) GRMs and the residual genome-wide sample of SNPs to construct background (B) GRMs. Statistics are shown for foreground G-BLUP analyses done using SNPs associated with *MP1*-*MP4*, *MP1*-*MP6*, *MP1*-*MP8*, and *MP1*-*MP10* (a single SNP was used for each locus included in the foreground). Genomic additive genetic variance (${\sigma}_G^2$) and narrow-sense heritability (${h}^2$) were estimated for the C0, C1R, and C2 populations. Genomic-estimated breeding values were estimated using either the whole-genome (standard), foreground (F), background (B), and F and B GRMs combined. Correlations ($r$) between phenotypic means ($\overline{Y}$) and GEBVs ($\hat{G}$) were estimated from 1000 cross-validation samples per analysis done using either the whole genome GRM or foreground and background GRMs individually (F or B) or in combination (F + B), where 80% of the individuals were randomly sampled for each analysis, ${\hat{r}}_G=r\left(\hat{G},\overline{Y}\right)$ is the correlation between $\hat{G}$ and $\overline{Y}$ from the standard analysis, $\overline{Y}$ are estimated marginal means (EMMs) for resistance phenotypes, $\hat{G}$ was estimated from the whole-genome GRM, ${\hat{r}}_F=r\left({\hat{G}}_F,\overline{Y}\right)$, ${\hat{G}}_F$ was estimated from a foreground GRM, ${\hat{r}}_B=r\left({\hat{G}}_B,\overline{Y}\right)$, ${\hat{G}}_B$ was estimated from a background GRM, ${\hat{r}}_{F+B}=r\left({\hat{G}}_F+{\hat{G}}_B,\overline{Y}\right)$, and ${\hat{G}}_F+{\hat{G}}_B$ was estimated from combined foreground and background GRMs

					Standard	*MP1*-*MP4*			*MP1*-*MP6*			*MP1*-*MP8*			*MP1*-*MP10*		
Cycle	Year	Location	${\hat{\sigma}}_G^2$	${\hat{h}}^2$	${\hat{r}}_G$	${\hat{r}}_F$	${\hat{r}}_B$	${\hat{r}}_{F+B}$	${\hat{r}}_F$	${\hat{r}}_B$	${\hat{r}}_{F+B}$	${\hat{r}}_F$	${\hat{r}}_B$	${\hat{r}}_{F+B}$	${\hat{r}}_F$	${\hat{r}}_B$	${\hat{r}}_{F+B}$
C0	2016	Davis	0.45	0.67	0.35	0.37	0.11	0.40	0.38	0.15	0.40	0.37	0.15	0.40	0.39	0.15	0.42
	2017	Irvine	0.39	0.47	0.63	0.53	0.25	0.58	0.57	0.16	0.58	0.66	0.14	0.60	0.59	0.29	0.60
		Combined	0.21	0.44	0.39	0.46	0.14	0.48	0.45	0.14	0.46	0.45	0.11	0.46	0.48	0.12	0.48
C1R	2022	Davis	0.38	0.30	0.40	0.23	0.26	0.37	0.24	0.27	0.36	0.30	0.24	0.40	0.33	0.26	0.41
		Salinas	0.36	0.17	0.33	0.23	0.22	0.31	0.29	0.19	0.34	0.31	0.19	0.36	0.31	0.20	0.37
		Combined	0.25	0.22	0.31	0.21	0.23	0.32	0.23	0.24	0.33	0.30	0.22	0.36	0.30	0.21	0.36
C2	2021	Davis	0.32	0.35	0.47	0.42	0.48	0.55	0.37	0.44	0.50	0.40	0.50	0.55	0.42	0.49	0.55
		Salinas	0.16	0.34	0.43	0.23	0.22	0.31	0.22	0.28	0.33	0.24	0.27	0.34	0.26	0.29	0.36
		Combined	0.24	0.40	0.42	0.27	0.35	0.42	0.28	0.38	0.43	0.26	0.36	0.41	0.30	0.38	0.44

**Figure 7:**
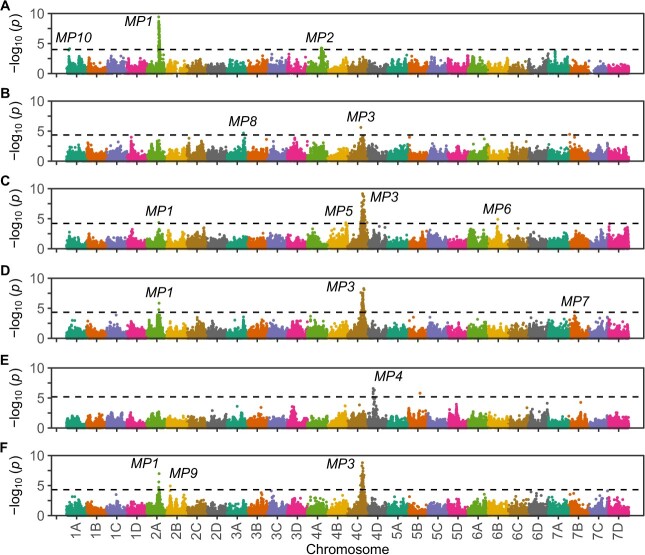
Manhattan plots displaying the statistical significance of SNPs associated with variation for resistance to *Macrophomina* observed among full-sib progeny in the C0, C1R, and C2 populations. The x-axis displays the physical addresses for 48 833 SNPs ascertained in the ‘Royal Royce’ reference genome (FaRR1). Manhattan plots are shown for analyses of: (A) 389 genotyped C0 individuals phenotyped in Davis and Irvine; (B) 1185 genotyped C1R individuals phenotyped in Davis; (C) 957 genotyped C1R individuals phenotyped in Salinas (D) C1R individuals across locations ($n=\mathrm{2,142}$); (E) 552 genotyped C2 individuals phenotyped in Davis; and (F) C0, C1R, and C2 individuals combined ($n=\mathrm{3,528}$). The dashed line demarcates the false discovery rate threshold for *p* = 0.05.

GEBVs ($\hat{G}$) and other statistics were estimated using standard G-BLUP, in addition to G-BLUP variations where different subsets of SNPs associated with 10 QTL for resistance to *Macrophomina* (*MP1*-*MP10*) were used for foreground selection (Table 2). The specific SNPs used for that purpose were identified by genome-wide association studies in the C0, C1R, and C2 populations (Fig. 7; Table 3). Their discovery, evidence of their validity, and the rationale for their inclusion in foreground selection are discussed in depth below.

**Table 3:** Statistics for SNPs associated with phenotypic variation for resistance to *Macrophomina* among $n$ selection cycle C0, C1R, or C2 individuals phenotyped in different locations and years. The frequency of the favorable allele (${\hat{f}}_{+}$), genotypic means for favorable allele homozygotes (${\overline{y}}_{+/+}$), heterozygotes (${\overline{y}}_{+/-}$), and unfavoarble allele homozygotes (${\overline{y}}_{-/-}$), additive ($\hat{a}$) and dominance ($\hat{d}$) effects, degree-of-dominance ($\hat{d}/\hat{a}$), chromosome numbers (CHR), and physical positions (bp) in the ‘Royal Royce’ genome are shown for the single-most significant SNP associated with *MP1* to *MP10* within and between populations and locations. The mean difference between heterozygotes and non-missing homozygotes ($\hat{b}$) was estimated when one of the homozygotes was missing. Favorable alleles were hypothesized for those SNPs by assuming $d/a\le 1$

Locus	SNP Marker^a^	SNP	+ Allele	CHR	${\hat{f}}_{+}$	Position (bp)	Cycle	Year	Location	$n$	${p}^2$	${\overline{y}}_{+/+}$	${\overline{y}}_{+/-}$	${\overline{y}}_{-/-}$	$\hat{a}$	$\hat{d}$	$\hat{d}$	$\hat{b}$	PVE^b^
*MP1*	AX-184504352	A/G	A	2A	0.65	15 419 803	C0	2016	Davis	381	7.20	3.95	4.47	4.78	−0.42	0.11	0.25	-	9.2
*MP1*	AX-184504352	A/G	A	2A	0.65	15 419 803	C0	Across	Across	381	9.42	4.22	4.62	4.86	−0.32	0.08	0.25	-	8.9
*MP1*	AX-184325782	A/G	A	2A	0.95	15 598 967	C1R	2022	Salinas	946	4.37	2.87	3.28	4.00	−0.57	−0.16	0.27	-	4.8
*MP1*	AX-184080864	T/G	T	2A	0.91	15 938 545	C1R	2022	Across	2139	5.85	2.64	3.22	3.41	−0.39	0.20	0.51	-	2.4
*MP1*	AX-184080864	T/G	T	2A	0.92	15 938 545	Across	Across	Across	3513	6.98	2.48	1.94	3.46	−0.49	−1.03	2.10	-	7.5
*MP2*	AX-184619882	T/G	T	4A	0.14	18 624 675	C0	Across	Across	389	4.26	3.92	4.24	4.58	−0.33	−0.01	0.03	-	7.1
*MP3*	AX-184790233	A/G	A	4C	0.25	20 480 448	C1R	2022	Davis	1180	4.21	2.14	2.37	2.72	−0.29	−0.06	0.21	-	2.3
*MP3*	AX-184438852	T/G	G	4C	0.75	19 926 202	C1R	2022	Salinas	955	9.15	2.68	2.64	3.18	−0.25	−0.29	1.16	-	4.4
*MP3*	AX-184775246	T/G	G	4C	0.81	21 502 509	C1R	2022	Across	2142	8.29	2.50	2.44	2.87	−0.19	−0.25	1.32	-	2.4
*MP3*	AX-184613479	A/G	G	4C	0.74	19 733 256	Across	Across	Across	3514	8.80	1.37	2.04	3.03	−0.83	−0.16	0.19	-	22.4
*MP4*	AX-184169019	A/G	G	4D	0.09	6 762 855	C2	2021	Davis	551	6.57	1.26	1.76	NA	-	-	-	−0.50	4.4
*MP5*	AX-184262201	T/C	C	4B	0.82	27 686 319	C1R	2022	Salinas	957	4.32	NA	2.66	3.06	-	-	-	−0.40	3.3
*MP6*	AX-184392125	A/G	A	6B	0.26	14 509 345	C0	2017	Irvine	581	5.47	3.58	4.10	4.57	−0.50	0.02	0.05	-	14.0
*MP6*	AX-184201573	A/G	G	6B	0.82	17 272 016	C1R	2022	Salinas	893	4.89	NA	2.78	2.98	-	-	-	−0.20	0.8
*MP7*	AX-123364954	T/G	T	7B	0.29	7 014 381	C1R	2022	Across	2126	4.40	2.34	2.61	2.87	−0.27	0.00	0.02	-	2.7
*MP8*	AX-184548398	A/G	A	3A	0.62	25 535 197	C1R	2022	Davis	1184	4.67	2.32	2.62	2.92	−0.30	0.00	0.00	-	3.3
*MP9*	AX-184337692	A/G	G	2B	0.35	4 537 820	Across	Across	Across	3527	4.92	2.07	2.81	3.16	−0.55	0.20	0.36	-	12.2
*MP10*	AX-166510834	A/G	A	1A	0.06	3 364 728	C0	2017	Irvine	571	6.97	NA	3.25	4.48	-	-	-	−1.23	20.7
*MP10*	AX-184369668	A/G	G	1A	0.92	3 484 664	C0	Across	Across	371	4.13	3.72	4.46	4.50	−0.39	0.35	0.90	-	7.1

a SNP markers used for survival and allele frequency change analyses are underlined.

b The percentage of the phenotypic variance explained by *MP1* to *MP10*-associated SNP.

c $p=-{\log}_{10}$ ($p$-value) for a test of the null hypothesis of no association between a SNP marker locus and ordinal *Macrophomina* resistance scores (1–5), where 1 = highly resistant (symptomless) and 5 = highly susceptible (dead).

The differences in narrow-sense heritability (${\hat{h}}^2$) and changes in EMM x GEBV distributions across cycles of phenotypic selection produced several insights (Table 2). First, the phenotypic differences we observed appeear to be moderately heritable. Our estimates of narrow-sense heritability (${\hat{h}}^2$) differed across cycles of selection and were lowest in the C1R population (Table 2). The slightly improved signal-to-noise ratios in the C0 or C2 populations were partly attributed to larger numbers of individuals at the phenotypic extremes (1 or 5), where replicate to replicate variation was lower. As noted earlier, when a significant number of individuals are dead (as in the C0 population) or symptomless (as in the C2 population), heritability increases because of the absence of phenotypic variation among these individuals.

Second, the correlations between phenotypic means (EMMs) and GEBVs were strongly positive, which suggested that genomic selection for resistance to *Macrophomina* should be as effective as phenotypic selection (Fig. 6; Table 2).

**Figure 8:**
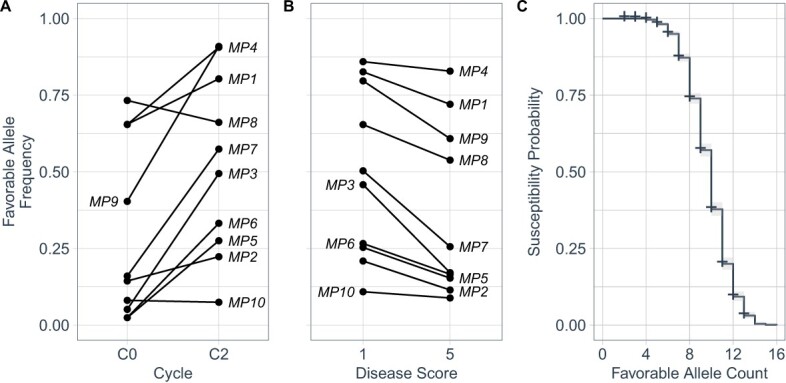
(A) Frequencies of SNPs associated with favorable *MP1* to *MP10* alleles in the C0 and C2 populations. The SNPs used in this analysis are identified in Table 3. (B) Frequencies of SNPs associated with favorable *MP1* to *MP10* alleles among highly resistant ($y=1$) and highly susceptible ($y=5$) individuals in the C1R and C2 populations combined. (C) The probability of susceptibility to *Macrophomina* estimated by survival analysis of individuals classified as resistant ($1\le y\le 2$) or susceptible ($2<y\le 5$) in the C1R and C2 populations. The independent variable was the number of favorable among predicted by SNP in linkage disequilibrium with the *MP1* to *MP10* loci. The dependent variable was the ordinal disease score.

Third, phenotypic selection drove GEBV means downward and appears to have greatly increased the frequencies of favorable alleles among several loci (Figs. 6–8). C2 individuals were predicted to have accumulated favorable alleles that were hidden and dispersed among C0 individuals (Fig. 6A and C). The complete inversion of the GEBV distributions between the C0 and C2 populations implied that phenotypically invisible favorable alleles transmitted by the parents of C1 full-sib families were effectively extracted, exposed by transgressive segregation among C1 and C1R hybrids (full-sib individuals), and further concentrated and accumulated among C2 hybrids (Fig. 6; Supplemental File S3). Interestingly, the lowest GEBVs in the C0 population were slightly greater than 3, well above the highly resistant range ($1.0\le G\le 2.0$); hence, none of the C0 genetic resources screened in our study had GEBVs in the resistant range, which makes the unforeseen genetic gains for resistance to *Macrophomina* even more extraordinary (Figs. 2–4 and6C).

The extremeness of the transgressive segregation observed among C1, C1R, and C2 progeny was not predicted by the cycle 0 EMM x GEBV distribution (Fig. 6A). This highlights the importance of perseverance in the face of extreme odds, the obvious necessity of hybridization, recombination, and accurate identification of transgressive multilocus *genotypes*, and the inherent difficulty of identifying sources of favorable alleles for certain complex traits (e.g., resistance to *Macrophomina*) in gene banks from the observed phenotypes or genomic-estimated breeding values of accessions alone, despite the power that can often be unlocked by genomic prediction in gene banks [80, 81]. While we are proponents of such approaches [77–79, 82], our retrospective analyses showed that *Macrophomina* resistance breeding values could not be accurately predicted from the training population phenotypes of diverse gene bank accessions alone (Fig. 6). Our genomic prediction study provides a cautionary tale about the effectiveness of genomic prediction for mining gene banks for favorable alleles. The predictive ability (${\hat{r}}_G$) estimates for resistance to *Macrophomina* were dismal when gene bank training population (C0) individuals were used to predict the breeding values of C1R (${\hat{r}}_G=0.15$) and C2 (${\hat{r}}_G=0.06$) individuals because of differences in population structure across cycles of selection and biases associated with strong selection within those populations, both of which are known to decrease the accuracy of genomic predictions between genetically divergent populations and breeds [83–86]. The reasons for the weak between-selection cycle genomic predictions in our study only became clear once we developed deeper insights into the genetics of resistance and could assess what drove the realized genetic gains from phenotypic selection (Table 1; Fig. 6).

Fourth, the breeding values of highly resistant individuals ($y=1$) were predicted with excellent accuracy in the C2 population (Fig. 6C). GEBV ranges were narrowest for the most resistant classes ($y=1$ and $y=2$), fanned out as phenotypic means for resistance score increased, and were widest for the highly susceptible class ($y=5$) in the C2 population (Fig. 6C). GEBVs of resistant individuals were more accurately predicted than GEBVs of susceptible individuals. The increase in noise among unselected individuals falling above a stringent truncation selection threshold (e.g., susceptible individuals with phenotypic means or GEBVs $\ge 1.5$) was inconsequential because accurately discriminating differences in susceptibility among unselected susceptible individuals has no bearing on the identification of highly resistant individuals (Fig. 6).

Lastly, the strongly right-skewed C2 distributions suggested that the purposeful addition of susceptible individuals (losers) to training populations could be essential for accurately predicting the breeding values of unphenotyped individuals [69]. This is an intriguing problem because individuals with symptoms (losers) became increasingly rare as selection progressed in our study (Fig. 6). We hypothesized that symptomless C2 individuals inherited different complements of favorable alleles (Fig. 8), which complicates genomic prediction because of the lack of phenotypic differences among different symptomless genotypes in the resistant tails of the phenotypic distributions. This threshold trait selection problem is precisely why MAS-enabled favorable allele stacking has significant merit (see below). We tested the hypothesis that different favorable allele combinations are found in symptomless individuals by searching the genomes of C0, C1R, and C2 individuals for genetic variants in linkage disequilibrium with loci affecting resistance to *Macrophomina*, assessing the complements of favorable alleles that they inherited, and estimating the probability of susceptibility using survival analysis (Fig. 7; Table 3).

### Survival analysis, the winner's curse, and testable favorable allele stacking solutions to the *Macrophomina* disease resistance breeding problem

The highly skewed, mirror image shapes of the C0 and C2 phenotypic distributions, rapid genetic gains between selection cycles 0 and 2, transgressive segregation in the C1, C1R, and C2 populations, and findings in a previous QTL mapping study [46] suggested that one or more large-effect loci might be associated with the phenotypic variation we observed for resistance to *Macrophomina* in strawberry (Figs. 2–5). To explore this, GWAS analyses were undertaken in the C0, C1R, and C2 populations (Fig. 7; Table 3; Supplemental Fig. S1-S2; Supplemental File S4-S6). Across populations, 3719 out of 4073 phenotyped individuals were genotyped with an Axiom 50 K SNP array [49] (Supplemental File S5). The physical positions of the SNPs on that array were ascertained by aligning SNP probe DNA sequences to the ‘Royal Royce’ reference genome (FaRR1) (https://phytozome-next.jgi.doe.gov/info/FxananassaRoyalRoyce_v1_0), which was annotated using the updated and corrected chromosome nomenclature described by Hardigan et al. [49] (Supplemental File S4).

We limited our GWAS analyses to SNPs with FaRR1 physical addresses validated by extensive comparative genetic mapping in octoploid populations (Supplemental File S4). This virtually eliminated false-positives caused by *in silico* assignments of SNPs to incorrect physical positions in the octoploid genome, most frequently on homoeologous chromosomes [49, 51]. Finally and importantly, we used the kinship matrix to correct for population structure and the cycle of selection (C0, C1R, and C2) as an independent variable (fixed effect) to correct for population strata, which was significant because artificial selection for resistance to *Macrophomina* profoundly altered allele frequencies and population structure (Figs. 2–3) [87, 88]. These corrections eliminated spurious population strata-associated genetic variants with signals exceeding the significance threshold, most of which were singletons not supported by haploblocks of SNPs in LD with an underlying causal locus.

**Figure 9:**
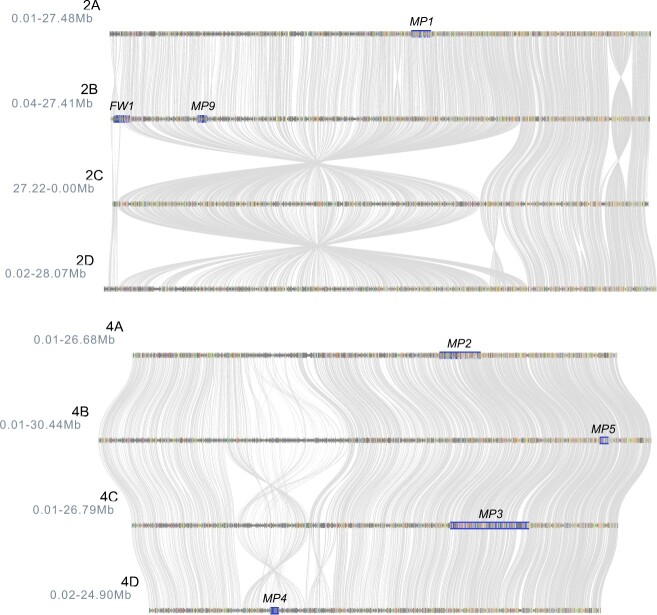
Genomic locations of *Macrophomina* resistance loci on chromosome 2 and 4 homoeologs in strawberry. The haploblocks predicted to harbor *MP1* on 2A, *FW1* and *MP9* on 2B, *MP2* on 4A, *MP5* on 4B,*MP3* on 4C, and *MP4* on 4D are highlighted below the locus names.

GWAS identified genetic variants associated with 10 loci affecting resistance to *Macrophomina* (Figs. 7–9; Table 3; Supplemental Fig. S1-S2; Supplemental File S6). Three (*MP1*–*MP3*) were previously identified in a Florida study, importantly where the pathogen isolate, segregating populations, and screening environment differed from ours [46]. The other seven (*MP4*–*MP10*) were identified in the present study. The effects of seven of these loci (*MP2* and *MP4*–10) were selection cycle or location specific (Fig. 7; Table 3). When GWAS was applied to phenotypes observed across cycles of selection and locations, statistically significant SNP associations were only identified for *MP1*, *MP3*, and *MP9* (Fig. 7F and9). *MP9* was the only one of those three that was not statistically significant in any of the selection cycle or location specific analyses, but was significant in the across-population analysis (Fig. 7A-E; Supplemental Fig. S1-S2). *MP9* physically mapped downstream of *FW1*, a Fusarium wilt resistance gene found near the upper telomere on chromosome 2B (Fig. 9) [76, 77]. The genomic segment harboring *FW1* and *MP9* appears to be inverted on homoeologous chromosome 2C (Fig. 9).

We recognize that the statistical support for some of these loci was comparatively weak (only slightly greater than the false-discovery rate threshold) and that the effects of some of these loci could have been overestimated as a consequence of the ‘winner's curse’ [89–91]; however, allele frequency changes between selection cycles 0 and 2 suggested that phenotypic selection increased the frequencies of their favorable (+) alleles, hereafter *MP1*^+^, *MP2*^+^, . . ., *MP1*0^+^ (Fig. 8; Supplemental File S7). To empirically assess the likelihood that they were targeted by phenotypic selection, we identified genetic variants (SNPs) associated with favorable and unfavorable alleles, estimated allele frequencies of *MP1* to *MP10*-associated SNPs among C0 and C2 individuals and among highly resistant ($y=1$) and highly susceptible ($y=5$) C1R and C2 individuals, and estimated the probability of susceptibility using survival or time-to-event analysis [52, 53], where favorable allele count was the independent variable, disease susceptibility was the dependent variable, and individuals were categorically classified as resistant ($1\le y\le 2$) or susceptible ($2<y\le 5$) for estimating the probability of survival (Fig. 8; Supplemental File S7).

We observed a statistically significant GWAS signal for a locus on chromosome 2A in the C0 population in Davis, the C1R population in Davis and Salinas, and across populations (Figs. 7A–B and9; Table 3; Supplemental Fig. S1-S2). We are confident that this is the *MP1* locus previously identified by Nelson et al. [46]. The statistically significant *MP1*-associated SNP identified in the C0 population (AX-184504352) physically mapped to chromosome 2A within 108 131 to 151 288 bp of the most significant SNPs (AX-89819605 and AX-166511224) identified by our GWAS reanalysis of AUDPC resistance phenotypes observed in the Florida study (Supplemental File S3). SNPs common to both studies were tiled on the 35 K and 50 K Axiom genotyping arrays [46, 49, 92], which facilitated the integration of unique SNPs by cross-referencing common SNPs (Supplementary Data S3).

The discovery of the *MP1* locus in the C0 population, or any other locus for that matter, was unexpected because of the strongly left-skewed phenotypic distributions, where approximately 90% of the C0 individuals were dead or near death (Fig. 2A–B). Using the most significant *MP1*-associated SNP identified in the C0 population (AX-184504352), the frequency of the favorable *MP1* allele (*MP1*^+^) was estimated to have increased from 0.65 in the C0 to 0.80 in the C2 population (Fig. 8A; Supplemental File S7). The additive effect of the AX-184504352 SNP ranged from −0.32 to −0.57 across populations (Table 3). The degree-of-dominance estimates for *MP1*-associated SNPs were in the incompletely dominant range ($0.25\le \hat{d}/\hat{a}\le 0.57$), apart from an over-dominant estimate in the across-population analysis ($\hat{d}/\hat{a}=2.10$), where the phenotypic mean for the heterozygote (${\overline{y}}_{+/-}=1.94$) was slightly less than the phenotypic mean for the favorable allele homozygote (${\overline{y}}_{+/+}=2.48$). The percentage of the phenotypic variance explained (PVE) by *MP1*-associated SNPs ranged from 7.5–9.6% (Table 3). The PVE estimates reported in Table 3 were from single locus analyses and are therefore non-additive. On the strength of the discovery of this locus in multiple populations and studies, we are confident that *MP1* is an important determinant of resistance to *Macrophomina* and warrants direct targeting by marker-assisted selection (MAS). The favorable allele appears to be present at a fairly high frequency among elite UC individuals, which was nonobvious from the phenotypes observed in the C0 population (Fig. 2 and6; Supplemental File S1).

We observed a statistically significant GWAS signal for a locus on chromosome 4A in the C0 population (Fig. 7A and9; Table 3; Supplemental Fig. S1-S2). We are confident that this is the *MP2* locus previously identified by Nelson et al. [46]. The statistically significant *MP2*-associated SNP identified in the C0 population (AX-184619882) physically mapped to chromosome 4A within 2 418 361 to 2 679 821 bp of the most significant SNPs (AX-123361466 and AX-166505860) identified by our GWAS reanalysis of AUDPC resistance phenotypes observed in the Florida study (Fig. 7A; Table 3; Supplemental Fig. S1-S3). Although the statistical significance of the *MP2*-associated SNP was lower than the *MP1*-associated SNP in the C0 population across locations, consistent with earlier findings [46], the respective additive effect and PVE estimates for *MP1* and *MP2* were similar ($\hat{a}=-0.32$ and $-0.33$ and PVE = 8.9 and 7.1%). Using the most significant *MP2*-associated SNP identified in our study (AX-184619882), the frequency of the favorable *MP2* allele (*MP2*^+^) was estimated to be 0.14 in the C0 and 0.22 in the C2 population; hence, this allele appears to be somewhat uncommon in diverse strawberry germplasm (Fig. 8; Supplemental File S7). Although statistically significant *MP2*-associated SNPs were not observed in the C1R or C2 populations or across populations, the frequency of the *MP2*^+^-associated SNP allele increased between the C0 and C2 generations and was greater among highly resistant ($y=1$) than highly susceptible ($y=5$) individuals in the C1R and C2 populations (Fig. 8). Despite the small PVE estimate for this locus in the combined analysis (1.8%), the effect of *MP2* has been observed in different populations and environments using different isolates of the pathogen. On the strength of these observations we concluded that *MP2* must be an important determinant of quantitative resistance to *Macrophomina*.

We observed a statistically significant GWAS signal for a locus on chromosome 4C in the C1R population phenotyped in Davis and Salinas and across populations (Fig. 7B-D and F and8; Table 3; Supplemental Fig. S1-S2). We are confident that this is the *MP3* locus previously identified by Nelson et al. [46]. The most significant *MP3*-associated SNP (AX-184438852) identified in the C1R population physically mapped to a position 131 639 bp distal to the most significant *MP3*-associated SNP (AX-184099604) identified by our reanalysis of AUDPC resistance phenotypes observed in the Florida study (Table 3; Supplemental Fig. S1-S3). The latter analysis was done by using the physical addresses of SNPs in the FaRR1 reference genome (Supplemental Fig. S3; Supplemental File S4). *MP2* and *MP3* were found in sytenic haploblocks on homoeologous chromosomes 4A and 4C; hence, the genes encoded by these QTL could be homoeologous (Fig. 9).

Using *MP3*-associated SNPs as proxies, we found that the favorable *MP3* allele was rare in diverse germplasm (${\hat{f}}_{+}$ ranged from 0.01 to 0.05) and appears to have been targeted by phenotypic selection for increased resistance to *Macrophomina* (Fig 8; Table 3; Supplemental File S7). The frequency of the favorable AX-184438852 allele increased from 0.05 in the C0 to 0.49 in the C2 population (Fig. 8; Supplemental File S7). Similarly, the frequency of the AX-184775246 allele increased from 0.01 in the C0 to 0.48 in the C2 population. Interestingly, we did not observe statistically significant *MP3*-associated SNPs in the C0 population, perhaps because of the rareness of the favorable allele among gene bank accessions. We cannot rule out the possibility that multiple favorable and unfavorable alleles segregated in our selected (C1, C1R, and C2) populations because we introduced allelic variation from several possible donors of favorable alleles for *MP3* and the other loci discovered in our study (Fig. 3; Supplemental File S1-S3). Our analyses of pedigree records and *MP3*-associated SNP marker genotypes suggests that Pacific Northwest cultivars (‘Totem’, ‘Tilikum’, ‘Linn’, and ‘Puget Reliance’) were sources of the favorable *MP3* alleles that were targeted by phenotypic selection. *MP3* explained greater percentages of the phenotypic variation for resistance than any of the other loci we identified, with estimates in the 18.5% to 22.4% range. The degree-of-dominance ranged from nearly additive ($\hat{d}/\hat{a}=0.19$) to slightly over-dominant ($\hat{d}/\hat{a}=1.32$) across populations and analyses. On the strength of these findings, we concluded that *MP3* is not only an important determinant but necessary for strong resistance to *Macrophomina*, common in Pacific Northwest germplasm, apparently common in *F. chiloensis* ecotypes native to western North America, and apparently uncommon in other germplasm resources.

Similar to *MP2*, the effects of *MP4* to *MP10* were mostly additive or incompletely dominant, weakly statistically significant, and selection cycle or location specific (Table 3; Fig. 7; Supplemental File S6; Supplemental Figure S1-S2). The strongest evidence we have that they could be legitimate determinants of resistance to *Macrophomina* were changes in the frequencies of SNPs in LD with causal loci (Fig. 8; Supplemental File S7). The favorable allele frequencies were greater for eight of the 10 loci in the C2 than the C0 population (Fig. 8A). When *MP1* to *MP10*-associated SNP allele frequencies were estimated across the C1R and C2 populations, we found that the favorable alleles were more frequent among highly resistant ($y=1$) than highly susceptible ($y=5$) individuals for every one of the 10 loci (Fig. 8). The allele frequency differences between resistant and susceptible groups were least dramatic for *MP10* and most dramatic for *MP3* and *MP7*–*MP9*. Our analyses show that initial frequencies of the favorable alleles across diverse germplasm resources (the C0 population) were widely different and rapidly increased (Supplemental File S7). Our analyses further show that resistance might be increased by driving certain favorable alleles to fixation because they were predicted to be additive or incompletely dominant (Table 3).

### Genomic selection weighted by large-effect loci

Once the large-effect loci were uncovered by GWAS, we explored the effectiveness of genomic selection schemes using different subsets of *MP1* to *MP10*-associated SNPs for foreground selection with and without the inclusion of residual genome-wide SNPs for background selection (Table 2; Fig. 6). Several conclusions emerged from these analyses. First, applying foreground selection to different subsets of SNPs in combination with residual genome-wide residual SNPs fairly consistently yielded the greatest predictive ability. Foreground-background genomic selection was superior to standard G-BLUP in seven of the nine analyses performed using the full complement of QTL-associated SNPs for foreground selection (last column in Table 2).

Second, starting with four loci (*MP1*–*MP4*) and sequentially targeting a larger number of foreground-selected loci negligibly increased predictive ability (Table 2). The predictive ability of the *MP1*–*MP4* subset was nearly as great as the *MP1*–*MP10* subset across cycles of selection and locations when foreground only and foreground-background genomic selection were applied. The effectiveness of the latter for predicting the breeding values of resistant individuals in the C2 population was outstanding (Fig. 6C). Nearly 100% of the GEBVs for individuals phenotypically classified as resistant ($1\le y\le 2$) were $\le 2$. Moreover, 100% of the individuals from the left tail of the GEBV distribution were symptomless and phenotypically classified as highly resistant ($y=1$). The effectiveness of applying genomic selection with direct pressure on specific QTL is perfectly illustrated by the dramatic changes observed amongst the C0, C1R, and C2 GEBV $\times$ EMM distributions for the *MP1*–*MP4* foreground-background genomic selection scheme (Fig. 6).

Third, genomic selection predictive ability was not dramatically different across cycles of selection or locations when foreground selection was applied to the full complement of loci (*MP1* through *MP10* in the foreground) with residual genome-wide SNPs in the background (Table 2). The predictive ability estimates from cross-validation for that selection scheme across locations were 0.48 in the C0, 0.36 in the C1R, and 0.44 in the C2 population.

Lastly, genomic selection predictive ability was lower for the C2 population in Salinas than Davis and was greater for standard G-BLUP than any of the foreground-background genomic selection schemes in Salinas. The reverse was true for the C2 population in Davis, where foreground and background selection combined were superior to standard G-BLUP. The patterns observed in the C2 population were especially important because C0 was an unselected founder generation and C1 and C1R were initial hybrid generations developed from selected founders (putative donors of favorable alleles) (Supplemental File S3). The most conservative path forward would be to genotype segregating populations with genome-wide DNA markers, which provides the flexibility of exploring genomic selection schemes with and without incorporating large-effect loci as fixed effects. This could be particularly important in populations where favorable allele frequencies and compositions are rapidly changing [93–96]. Our results suggest, however, that direct marker-assisted stacking of favorable *MP1* to *MP10* alleles, in some combination (Fig. 8), might ultimately prove to be as effective as genomic selection with or without the incorporation of large-effect loci (Fig. 6; Table 2).

### Defense-related genes found in haploblocks predicted to harbor *Macrophomina* resistance QTL

To identify and catalog defense-related genes in close proximity to SNPs associated with the *Macrophomina* resistance QTL identified in our study, search windows in the ‘Royal Royce’ reference genome (FaRR1) were defined by using a $2\times -{\log}_{10}$(*p*-value) decrease in the statistical significance of SNPs upstream and downstream of the most significant SNPs identified by GWAS (Table 3; Fig. 7,9; Supplemental File S8; https://phytozome-next.jgi.doe.gov/info/FxananassaRoyalRoyce_v1_0). This process was repeated to define search windows for QTL identified in different populations where the most significant SNP differed (*MP1*, *MP3*, *MP6*, and *MP10*). Our search identified 965 genes across the 10 haploblocks, of which 205 had motifs or functions associated with disease resistance in plants (Supplemental File S8). These were lumped into qualitative (gene-for-gene) and broader quantitative (polygenic) resistance groups [2, 4, 24, 25]. While classic disease resistance (*R*) genes, e.g, genes with nucleotide binding site (NBS) and leucine-rich repeat (LRR) or serine/threonine protein kinase (S/TPK) domains, were cataloged for completeness (Supplemental File S8), they are presumably less likely candidates for the QTL identified in our study than other classes of defense-related genes [2, 97]. We identified 139 genes with defense-related functions that could conceivably be involved in quantitative resistance to plant diseases, especially but not limited to those caused by necrotrophic pathogens [2, 4, 24, 25]. These included cupredoxin, AP2 and GRAS family transcription factors, proteases in the subtilase and papain-like cysteine peptidase classes, and others (see Supplemental File S8 for details). Their functional annotations in the ‘Royal Royce’ genome, best BLAST hits to Arabidopsis genes, and physical positions of the most significant and haploblock-boundary SNPs are tabulated in Supplement File S8.

The sheer number of candidate genes found in our search, inherent biases associated with gene annotation ontology and classification [98], and statistical uncertainty associated with the physical locations and effects of the underlying QTL accentuate the challenge ahead in studies undertaken to identify causal genes and mutations underlying *MP1–MP10* (Fig. 7 and9). Their identification obviously does not preclude effective predictive breeding for resistance to this pathogen, but would empower studies to resolve haplotypes and identify genetic variants for more accurately predicting and tracking favorable and unfavorable alleles across populations. The merit of identifying genes underlying some of these QTL seems minimal, especially without extensive validation of their effects [99]. *Macrophomina* resistance breeding values can be predicted with sufficient accuracy in segregating populations with genome-wide information without knowing the genes (Fig. 6; Table 2). The resolution of haplotypes and identification of causal loci and mutations or highly predictive genetic variants in LD with the causal loci, however, has significant merit [100, 101]. The two QTL that most obviously warrant a search for the underlying causal genes and mutations are *MP1* and *MP3*. These QTL had the largest effects, have been validated in multiple populations and environments, and are predicted to be necessary for resistance (Table 3; Fig. 7) [46].

## Discussion

The emergence of a quick and effective solution to the *Macrophomina* disease resistance breeding problem seemed improbable after our phenotypic screening of strawberry gene bank resources turned up nothing that was unambiguously highly resistant (Fig. 2A-B and6; Supplemental File S1). We speculated that our screening protocols had been overly harsh, and they may have been; however, that harshness turned out to have been critical for uncovering favorable alleles, accelerating genetic gains, and unraveling the genetic mysteries that initially eluded us (Fig. 6; Supplemental File S1). *MP1*, *MP2*, and *MP3* had not yet been discovered [46] and the rareness of resistant individuals in our gene bank screening studies suggested that a straightforward formulaic solution to the *Macrophomina* disease resistance breeding problem was unlikely (Fig. 2A–B). While our initial findings painted a bleak picture, they were not wholly unexpected from the ‘black box’ genetic architecture commonly observed for quantitative resistance to generalist necrotrophic pathogens like *Macrophomina* [2, 4–6, 11, 17 ].

Our early pessimism was soon replaced by cautious optimism before being erased altogether as the phenotypic distributions shifted shape and inverted skewness across cycles of selection (Fig. 2,6). That early pessimism, which did not sway us from forging ahead with phenotypic selection, was influenced by findings in soybean and other agriculturally important plants where the genetics of resistance to *Macrophomina* was known to be complex and does not appear to be conferred by qualitative (gene-for-gene) resistance [2, 13, 14, 17, 102]. The resistance of strawberry to *Macrophomina* is not governed by gene-for-gene resistance either, nor was that ever hypothesized; however, the discovery of multiple large-effect loci was not hypothesized by us either (Fig. 7; Table 3). We initially predicted that genetic variation for resistance to this pathogen was limited, which implied that long-term selection would be necessary to develop resistant cultivars and that strong resistance *might not* be attainable [48]. We were wrong on both counts (Table 1; Fig. 6).

Although the positive correlation between heat and drought stress and disease development and severity has only been anecdotally established for *Macrophomina* in strawberry [18, 19, 34, 37, 38, 40], a cause–effect relationship seems plausible and has been postulated for *Macrophomina*-caused diseases in other plants [23, 103–107]. The strawberry *Macrophomina* problem coincidentally surfaced in California, Florida, and elsewhere in the wake of the methyl bromide phase-out and appears to have been aggravated by an increase in heat and drought stress attributed to climate change [20, 35, 38, 41, 108, 109]. The hypothesized impact of climate change-associated abiotic stresses on the emergence of this disease seems tenable because *Macrophomina* either never surfaced or was uncommon and undetected in organic production in California over the half century preceding the methyl bromide phase-out in 2005 [20, 21, 35, 36]. The synergistic effects of climate change and changes in fumigation practices are undoubtedly responsible for the recency of the *Marcrophomina* disease problem in strawberry. The incidence and severity of *Macrophomina*-caused disease are bound to increase in strawberry as the incidence and severity of heat and drought stress increase across the globe [23, 103].

We found that phenotypic screening under heat and drought stress was critical for identifying sources of favorable alleles, decreasing the probability of false positives (plants declared to be resistant that are not), accurately assessing the disease reactions of cultivars, and identifying highly resistant individuals (Fig. 4 and 6). We contend that the disease reactions and resistance claims made for cultivars phenotyped under non-stress conditions are spurious and mislead farmers that depend on accurate information for risk mitigation. False disease claims arise when susceptible cultivars are reported to be symptomless or to have mild symptoms under non-stress conditions where genetic differences among cultivars cannot be uncovered or accurately estimated. The importance of phenotyping reactions to this disease under stress conditions cannot be overstated. Accurate phenotyping and phenotypic selection under high summer temperatures and induced drought stress were essential for driving the genetic gains and discoveries reported in our study.

The resistance of strawberry to *Macrophomina* is genetically complex, perplexing, and curiously predicable. On the one hand, several large-effect loci appear to play a prominent role in the reaction of strawberry to *Macrophomina* (Fig. 7; Table 3) [46], a result predicted by transgressive segregation and genetic gains from phenotypic selection (Figs. 2–4; Table 1). On the other hand, the weak to moderate narrow-sense heritability of resistance and the QTL quagmire uncovered by association studies created considerable uncertainty and left many questions unanswered (Fig. 7; Tables 2-3). How certain are we that the 10 loci we identified are valid and sufficient for solving the *Macrophomina* disease resistance breeding problem in strawberry? Even if these 10 loci are validated, is a 10-locus marker-assisted stacking strategy practical, sensible, or necessary?

We propose here that stacking favorable alleles among five or fewer loci should be sufficient for acquiring strong resistance to this pathogen (Fig. 8). We discovered that the favorable alleles needed for maximizing resistance had previously not been aggregated and accumulated through hybridization, recombination, and selection in strawberry cultivars, which makes perfect sense when you consider that the first reports of this disease have only emerged in the last 20 years, that breeding for resistance to this pathogen has been underway for less than a decade, and that insights into the genetics of resistance were virtually non-existent when our studies were initiated in 2015 [18–21, 27, 35–38, 46]. Transgressive segregation, the driving force behind our genetic gains [63–66], implied that elite and exotic donors of favorable alleles (C0 parents of C1 and C1R progeny and C1 parents of C2 progeny) were accurately identified and that those alleles were rapidly accumulated (Figs. 2–4 and6; Supplemental File S3) without an understanding of or even a need to understand the genetic architecture of resistance [5, 65].

We drew parallels between the fabled and once controversial ‘hopeful monsters’ of evolutionary biology—which arise from sudden, discontinuous genetic changes in hybrids—to the extreme transgressive segregates that arose among hybrids between elite cultivars and exotic donors of favorable alleles in our study [65–67, 110]. The origin of novelty and sudden appearance of previously unobserved phenotypes (hopeful monsters) has not only been long accepted in plant breeding, but essential, expected, and sought after [65]. Indeed, the creation and identification of transgressive segregates carrying previously non-existent or uncommon combinations of favorable alleles have been the principal drivers of genetic gains from artificial selection in domesticated plants [65]. The sudden, discontinuous genetic changes we observed were more extreme than predicted from the murky phenotypes of the founders (C0 parents) and appear to have been driven by the segregation of multiple small- to large-effect loci and the accumulation of favorable alleles among them (Fig. 7-8).

The parents of our C1, C1R, and C2 populations were selected before any of the loci described here had been identified (Table 3; Fig. 7; Supplemental Files S1 andS3) [46]. The insights we gained from analyses of allele frequency changes and variable effects of those loci across cycles of selection later shed light on why phenotypically obvious sources of resistance to *Macrophomina* were found to be virtually non-existent in strawberry gene banks. We discovered that a critical number of favorable alleles had to be accumulated or ‘stacked’ before an individual acquired strong resistance (Fig. 2-5 and6-8; Supplemental File S1). The exact number and combinations are not completely clear because resistance appears to have been achieved by multiple combinations of favorable alleles among loci with variable effects. Moreover, the additive and dominance effects and degree-of-dominance of the underlying loci still need to be carefully estimated in a more balanced multilocus statistical genetic framework, which did not exist in our rapidly evolving selected populations.

We did not originally envision or intentionally set out to solve the *Macrophomina* disease resistance breeding problem by hunting down QTL, a practice that more often than not has been futile, especially when the QTL effects are population specific, statistically weak, or overestimated [89–91, 93–95, 99, 111, 112]. However, as each cycle of selection unfolded, previously unidentified QTL were exposed, and others reappeared and were validated by transgressive segregation and allele frequency changes [63–66]. This was not an academic exercise where the discovered QTL were destined to collect dust on a “library shelf” or irrationally inflate expectations before eventually wallowing in a “trough of disillusionment” [99, 111, 112]. To the contrary, we had already achieved our breeding goal (strong resistance to *Macrophomina*) by applying phenotypic selection before tackling genome-wide searches for associations between resistance phenotypes and causal loci that were designed to shed light on what might have driven the rapid and dramatic genetic gains we observed (Fig. 6). We present evidence that our selection experiments extracted favorable alleles previously hidden in diverse elite and exotic genetic resources, targeted multiple loci, profoundly changed the frequencies of favorable alleles among those loci, and created novel genotypes (highly resistant transgressive segregates) carrying stacks of favorable alleles that were either previously non-existent or exceedingly rare in strawberry (Fig. 8) [63–66]. 

The loci uncovered in our phenotypic selection studies, if nothing else, created testable hypotheses about the complements of favorable alleles that are necessary and possibly sufficient for acquiring strong resistance to *Macrophomina* in strawberry. We hypothesize that *MP1*^+^, *MP3*^+^, and *MP9*^+^ are necessary for resistance and that one or more additional favorable alleles could be necessary for maximizing resistance. We are most confident that resistance to this pathogen can be predictably and reliably increased by stacking *MP1*^+^, *MP3*^+^, *MP4*^+^, *MP5*^+^, and *MP9*^+^ alleles. We are less confident that the other five favorable alleles are necessary for resistance or that they need to be directly targeted by MAS to develop resistant cultivars (Table 3). We are well down the path of validating the effects of these QTL in populations developed for phenotypic and genomic selection. Our analyses predict that genetic gains can be maximized by incorporating the fixed effects of specific large-effect QTL in genomic predictions [69]. The importance of individual QTL can be fluidly reassessed by GWAS, survival analysis, and analyses of the effects of selection on favorable allele frequencies, as was done in the present study (Fig. 8). Moreover, the merits of targeting specific QTL can be continually reevaluated and updated as training populations evolve [48, 69]. Despite the uncertainties and ambiguities associated with some of the loci identified in our study, survival analysis [52, 53] using favorable allele counts suggested that resistance can be predictably increased by stacking different combinations of favorable alleles via marker-assisted selection (Fig. 8). Our study laid the foundation for pressure testing that proposal, in addition to showing that genomic selection *per se* is a viable solution to the *Macrophomina* disease resistance breeding problem in strawberry.

## Materials and methods

### Plant Material & Propagation

The germplasm accessions (C0 population) phenotyped in our study were 853 asexually propagated *F.*$\times$*ananassa*, *F. chiloensis*, and *F. virginiana* individuals preserved in the UC Davis (UCD) Strawberry Germplasm Collection (SGC) and United States Department of Agriculture (USDA) National Plant Germplasm System (NPGS) collections (https://www.ars.usda.gov/). We acquired ‘mother’ plants for 265 of these individuals from the USDA NPGS National Clonal Germplasm Repository in Corvallis, Oregon (https://www.ars.usda.gov/pacific-west-area/corvallis-or/). The other 588 were among holdings in the UC Davis Strawberry Germplasm Collection as of February 1, 2015. C0 individuals were preserved and annually propagated from mother plant stolons at the Wolkskill Experiment Orchard (WEO), Winters, CA over the course of our studies. UCD and USDA identification and plant introduction numbers, aliases, pedigrees, and passport information for C0 population individuals ($n=853$) are tabulated in Supplemental File S1). The C0 population was comprised of 788 *F.*$\times$*ananassa* cultivars and other hybrids, 39 *F. chiloensis* ecotypes, and 36 *F. virginiana* ecotypes (Supplemental File S1). The daughter plants (bare-root clones) of C0 individuals were produced from mother plants grown at a low-elevation location (41 m; Winter, CA) for the spring-planted 2016 study and a high-elevation location (1284 m; Dorris, CA) for the fall-planted 2016 to 2017 study.

Several full-sib families were developed for the C1, C1R, and C2 populations phenotyped in our studies. The parents and full-sib family identification numbers for these populations are tabulated in Supplement File S3. The parents were grown in UCD greenhouses from early fall to late spring for manual emasculation of female parents, hybridization, and full-sib family seed production. Fruit harvested from female parent plants were macerated in a pectinase solution (0.6 g/L) to separate seeds (achenes) from receptacles. Seeds were scarified in a 36 N sulfuric acid solution for 16 minutes, rinsed in water, dried on blotter paper, germinated in kiln-dried artificial media (two parts vermiculite to one part sand) at room temperature, transplanted into peat pellets, and grown in a shade house in Winters, CA before being artificially inoculated with the pathogen and transplanted to the field in late October or early November of each year. Seedlings were grown in the shadehouse for three to five months before transplanting. The bare-root plants of check cultivars were produced in Doriss, CA as described above for C0 germplasm accessions, inoculated with the pathogen, and transplanted alongside C1, C1R, and C2 seedlings in each location. We grew and phenotyped 2211 C1, 2220 C1R, and 1001 C2 individuals.

### Pathogen Source Material & Inoculum Preparation

The *M. phaseolina* isolate used for artificial inoculation of bare-root plants and seedlings in our 2016 to 2020 studies (‘GL1310’) was collected in 2007 from an infected strawberry plant in Orange County, CA. The isolate used in our 2021 study (‘Mp11–12’) was collected from an infected strawberry plant in Santa Barbara, CA in 2011 [113, 114]. The fungus was grown on potato dextrose agar (PDA) plates (60 × 15 mm) for 1 week in the dark at room temperature. Small squares (1.5 × 1.5 mm) of PDA were cut from those plates and replated on PDA plates (90 × 15 mm) amended with tetracycline (50 mg/l). These were grown in the dark at room temperature for 3–4 weeks. The PDA was blended with sterile deionized (DI) water in a Waring Pro blender Model 51BL23 (Waring Commercial, Torrington, CT 06790). We added one liter of water to the blended PDA material from seven to eight plates, checked the spore concentrations with a haemocytometer, and added more water as needed to reach a concentration of approximately 7 × 10^6^ sclerotia/ml. We produced appoximately 20 l of inoculum for each study from 150 90 × 15 mm PDA plates.

### Field studies and phenotyping

Our field experiments were conducted using identical pathogen inoculation, planting, and phenotyping protocols across locations and years. C0 population bare-root clones were planted March, 2016 at the UC Davis Plant Pathology Farm (Davis, CA) and October, 2016 at the UC Agricultural and Natural Resources South Coast Research and Extension Center (Irvine, CA). C1 population seedlings and check cultivars were planted November, 2018 at the UC Davis Plant Pathology Farm. C1R population seedlings and check cultivars were planted November, 2021 at the UC Davis Plant Pathology Farm and Garcia Farm (Salinas, CA) in cooperation with the USDA Agricultural Research Service Crop Improvement and Protection Research (Salinas, CA). C2 population seedlings and check cultivars were planted November, 2020 at the UC Davis Plant Pathology Farm and Garcia Farm. The roots of seedlings and bare-root plants were submersed in inoculum solution for 5 to 7 minutes. Before transplanting.

We initiated phenotyping in the precipitation-free months of June and July in Davis, CA when daily temperatures were increasing, drought stress could be induced by decreasing irrigation by 50% or more, and disease symptoms began appearing. The daily high temperatures were in the 27–42 -°C range, whereas the daily low temperatures were in the 8°C to 17 °C range over those months with zero to near zero precipitation (https://www.weather.gov/wrh/climate). Ordinal scores from 1 to 5 were assigned according to the severity of the symptoms, where 1, highly resistant (symptomless) and 5, highly susceptible (dead), as shown in Fig. 1 and described by Koike et al. [21]. Visual symptoms were observed and ordinal scores were recorded once per week in the early cycles of selection (C0 and C1) and decreased to every other week for the later cycles of selection (C1R and C2) as we gained experience with symptom progression trends and as the frequency of resistant individuals changed over cycles of selection. We used the incidence and severity of symptoms and phenotypic distributions of ordinal scores as guides for terminating phenotyping. Statistical analyses were applied to ordinal scores recorded on the last time point in July in each study, apart from the C1R population study in Salinas, which was phenotyped through October.

The soils are at our study locations were classified as Yolo loam (Davis), San Emigdio fine sandy loam (Irvine), and Chualar loam (Salinas) (https://websoilsurvey.sc.egov.usda.gov/). Strawberries had not been grown on any of the fields selected for our studies. The field used for the 2016 study in Davis was not fumigated. The fields used for the 2016–17, 2018–19, 2020–21, and 2021–22 studies in Davis with flat-fumigated approximately three months before planting with Pic-Clor 60 (Cardinal Professional Products, Woodland, CA) at a rate of 560.426 kg/ha. The fumigated fields were sealed with a totally impermeable film tarp for at least one week. The fields used for the 2020–21 and 2021–22 studies in Salinas were drip-fumigated with Triform 80 or Pic60 at a rate of 140.3 l/ha. Drip irrigation lines and black plastic mulch were installed in each field before planting. Plant were grown in 15.25 cm high single-row raised beds in Davis and Irvine with 30.5 cm spacing between plants and 76.2 cm spacing between beds center-to-center. Plants were grown in 30.5 cm high two-row raised beds in Salinas with 40.6 cm spacing between plants and 101.6 cm spacing between beds center-to-center. Our field experiments were sub-surface drip-irrigated as needed to maintain adequate soil moisture and injection fertilized through the drip irrigation system. Approximately 169 to 198 kg/ha of nitrogen was applied over the growing seasons.

We used randomized complete blocks (RCB) experiment designs with four single-clone replicates (four complete blocks) for the C0 population study. There were 576 entries/block (2304 experimental units) in the 2016 Davis study and 960 entries/block (3840 experimental units) in the 2016–17 Irvine study. We used augmented RCB experiment designs for the other studies with unreplicated seedlings (full-sib progeny) and four single-clone replicates per check cultivar. The number of check cultivars ranged from four for C1R and C2 to 17 for C1. The number of unreplicated seedlings per study were 2211 (C1 population Davis), 1260 (C1R population Davis), 960 (C1R population Salinas), 560 (C2 population Davis), and 441 (C2 population Salinas). The randomization plans for entries within blocks were generated using *design.rcbd()* or *design.dau()* function in the R package *agricolae* [115].

### Statistical analyses

Linear mixed models (LMMs) were modeled and analysed using *lme4::lmer()* [116]. Clone-based broad-sense heritability (${H}^2$) was calculated as:

$$ {H}^2=\frac{V_G}{V_G+\frac{V_R}{h}} $$

where ${V}_G$ is the variance associated with the accession, ${V}_R$ is the residual variance, and $h$ is the harmonic mean of the number of replicates per accession, calculated using *pysch::harmonic.mean()* [117]. Cross-year broad-sense heritability was calculated as:

$$ {H}^2=\frac{V_G}{V_G+\frac{V_{G\cdotp T}}{t}+\frac{V_R}{t\cdotp h}} $$

where ${V}_{G\cdotp T}$ is the variance associated with the genotype-by-experiment interaction, and $t$ is the number of experiments. Family-based heritabilities were calculated as:

$$ {H}_F^2=\frac{V_F}{V_F+\frac{V_{F\cdotp L}}{2}+\frac{V_R}{2\cdotp h}} $$

where ${V}_F$ is the variance associated with full-sib families, ${V}_{F\cdotp L}$ is the variance associated with family-by-environment interaction (across the Davis and Salinas locations tested), and $h$ is the harmonic mean of the number of individuals (full-sibs) in each family.

Estimated-marginal means (EMMs) were calculated for replicated clonal accessions using *emmeans::emmeans()*, correcting for the effect of block in randomized complete block experiment designs [118–120].

### SNP genotyping

DNA was isolated from freshly emerged leaves from greenhouse or field grown plants. Leaf tissue was placed into 1.1 ml tubes, freeze-dried in a Benchtop Pro (VirTis SP Scientific, Stone Bridge, NY), and ground using stainless steel beads in a Mini 1600 (SPEX Sample Prep, Metuchen, NJ). Genomic DNA (gDNA) was extracted from powdered leaf samples using the E-Z 96 Plant DNA Kit (Omega Bio-Tek, Norcross, GA, USA) according to the manufacturer’s instructions. To enhance the quality of the DNA and reduce polysaccharide carry through, the protocol was modified with a Proteinase K treatment, a separate RNase treatment, an additional spin, and heated incubation steps during elution. DNA quantification was performed using Quantiflor dye (Promega, Madison, WI) on a Synergy HTX (Biotek, Winooski, VT).

The individuals in our studies were genotyped with a 50 K Axiom SNP array [49] on a GeneTitan HT Microarray System by Affymetrix (Santa Clara, CA) using gDNA samples that passed quality and quantity control standards. SNP genotypes were automatically called using Affymetrix Axiom Analysis Suite software (v1.1.1.66, Affymetrix, Santa Clara, CA). Samples with call rates greater than 89% were analyzed. The genomic relationship matrix (G) among individuals was estimated using the function ‘*A.mat)’* in the R package *‘rrBLUP’* using a minor allele frequency cutoff of 0.05 (min.MAP = 0.05) and a maximum missing data cutoff of 0.5 (max.missing = 0.5) with imputation of missing data (return.imputed = TRUE)’ [121].

### Genome-wide association studies

We used the R package *rrBLUP* and the *rrBLUP::GWAS()* function for genome-wide association studies [121] using the ‘Royal Royce’ genome (FaRR1) as a physical reference (https://phytozome-next.jgi.doe.gov/info/FxananassaRoyalRoyce_v1_0) and 50 K Axiom array SNP genotypes as independent variables [49]. SNP markers on the 50 K Axiom array were physically anchored to the FaRR1 genome (Supplemental File S4). The physical positions of SNPs used in our nalyses were validated by extensive genetic mapping. SNPs that genetically mapped to homoeologous chromosomes different from those originally assigned were physically remapped by BLAST searches to the correct chromosomes. We limited our GWAS analyses to SNPs with chromosome (physical position) assignments validated by genetic mapping (Supplemental File S4-S5).

GWAS was applied to the estimated marginal means for disease score observed among C0, C1R, and C2 population individuals within and across locations and across populations. The genomic relationship matrix (G) estimated from 50 K Axiom array SNP marker genotypes was used to correct for population structure. We used population as a fixed effect for the across-population GWAS. The genomic inflation factors ($\lambda$) for analyses of C0, C1R, and C2 populations ranged from 0.98–1.01, 0.91–0.96, and 0.96–1.01, respectively, The genomic inflation factor for the across-population analysis was 0.92. False discovery rate (FDR) thresholds for *p* = 0.05 were calculated using the R package *qvalue* [121–123]. The percent of the phenotypic variance explained (PVE%) by the most significant QTL-associated SNPs were estimated the semivariance method [124].

We searched annotations in the FaRR1 genome within haplobocks flanking the most significant QTL-associated SNPs to identify and catalog genes in close proximity to *MP1*-*MP10*. We first defined peak regions using a 2-log drop strategy: for each locus searching up- and down-stream from the peak SNP until reaching a SNP with significance $2\times -{\log}_{10}(p$-value) lower. To account for potential over-stringency of this method, we extended peak regions with +/− 250 kb buffer. For loci which were found in multiple years this process was done for each instance. We then extracted gene annotations in the FaRR1 genome in these regions and for counting removed duplicates from loci discovered more than once, resulting in the reported 965 genes within MP1–MP10 significance intervals (https://doi.org/10.25338/B8TP7G;Supplemental File S8).

To identify potentially homoeologous QTL regions, we first inferred strawberry homeologs with the GENESPACE pipeline [125], which uses a combination of protein sequence similarity and gene synteny to infer homologs. The ‘Royal Royce’ genome assembly (https://phytozome-next.jgi.doe.gov/info/FxananassaRoyalRoyce_v1_0) A, B, C, and D subgenomes were separated and compared. The locations of GENESPACE-inferred homeologs were then visualized using the graphics tools of JCVI [126], with significant regions for each QTL identified via flanking genes and visualized by blue bars.

GWAS was applied to previously published data from a study in Florida [46] using the aforementioned R packages and statistical methods. Genotypic and phenotypic data were extracted from Supplemental Files S1-S3 of Nelson et al. [46]. We reanalyzed their data using GWAS so that the physical positions of *MP1*, *MP2*, and *MP3*-associated SNPs could be identified in FaRR1 genome identified using SNP markers shared between the 35 K SNP array used in their study [92, 127] and the 50 K SNP array used in our study [49] (Supplemental File S4).

### Genomic prediction analyses

Genomic prediction experiments were conducted using the *sommer* v4.3.1 R package with *sommer::mmer()* [128] within selection cycle and within and among locations. Our CV strategy was 80/20 random CV where a random 20% of the phenotypic observations (test) were withheld from the model and predicted using the remaining 80% (train). We performed 100 iterations for each of four models and recorded the predictive ability—the Pearson correlation between GEBV and EMM in the testing set—for each iteration. This approach resulted in a total of 45 experiments (5 models $\times$ 3 cycles $\times$ 3 between and among location combinations).

The first approach is standard G-BLUP, a linear mixed model with a single random effect:

(1) \begin{equation*} \overline{Y}=\mu +\sim \mathcal{N}\left(0,{\mathbf{G}}_G{\sigma}_G^2\right)+\sim \mathcal{N}\left(0,\mathbf{I}{\sigma}_R^2\right) \end{equation*}

where $\overline{Y}$ are the phenotypic resistance scores, $\mu$ is the population mean, ${\mathbf{G}}_G$ is the standard GRM calculated from all SNPs, ${\sigma}_G^2$ is the additive genomic variance, and ${\sigma}_R^2$ is the residual variance.

The other three approaches incorporated foreground and background random effects associated with the significant loci discovered by GWAS ($F$ for foreground selection) and the remaining SNPs ($B$ for background selection):

(2) \begin{equation*} \overline{Y}=\mu +\sim \mathcal{N}\left(0,{\mathbf{G}}_F{\sigma}_{G_F}^2\right)+\sim \mathcal{N}\left(0,{\mathbf{G}}_B{\sigma}_{G_B}^2\right)+\sim \mathcal{N}\left(0,\mathbf{I}{\sigma}_R^2\right) \end{equation*}

where ${\mathbf{G}}_F$ is the additive GRM calculated from the foreground SNPs, i.e., *MP1–4*, *MP1–6*, *MP1–8*, and *MP1–10*, for approaches 2–5 respectively, ${\mathbf{G}}_B$ is the standard GRM calculated from remaining background SNPs, specifically not MP1–4, *MP1–6*, *MP1–8*, or *MP1–10*, ${\sigma}_{G_F}^2$ is the additive genomic variance associated with the foreground SNPs, ${\sigma}_{G_B}^2$ is the additive genomic variance associated with the background SNPs, and ${\sigma}_R^2$ is the residual variance.

## Supplementary Material

Web_Material_uhad289
