# Supplementary figures and images for: Transgressive segregation, hopeful monsters, and phenotypic selection drove rapid genetic gains and breakthroughs in predictive breeding for quantitative resistance to Macrophomina in strawberry
Source: Hortic Res. 2024 Jan 3;11(2):uhad289. doi: 10.1093/hr/uhad289 (PMC10939388; doi:10.1093/hr/uhad289)

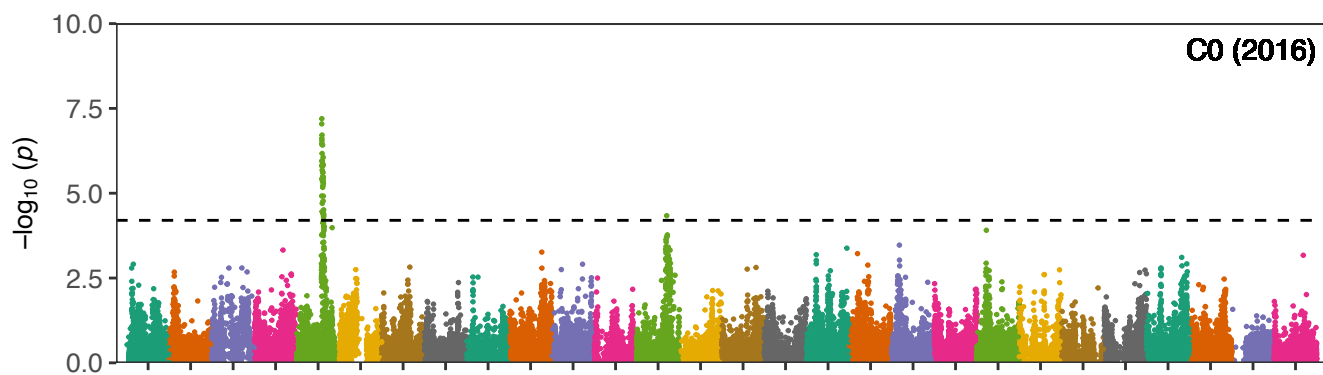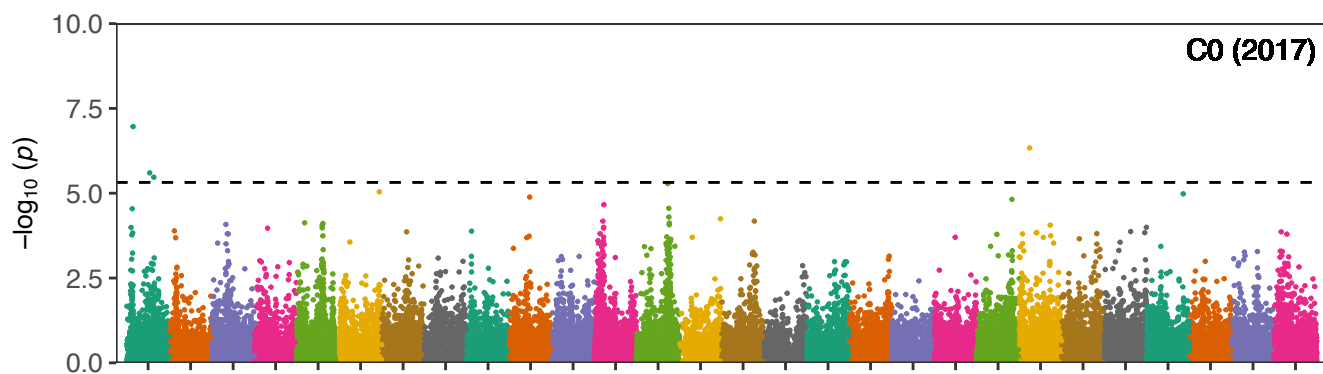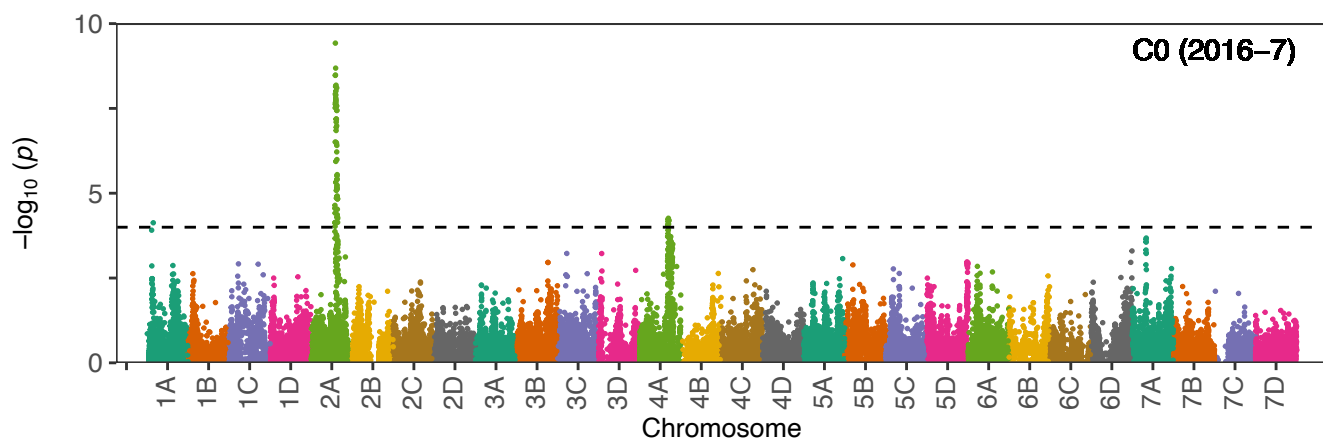

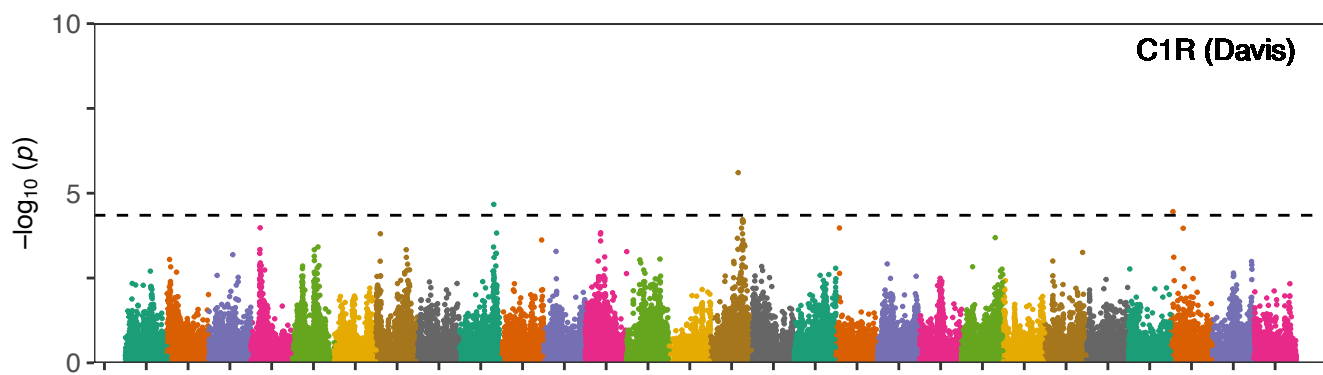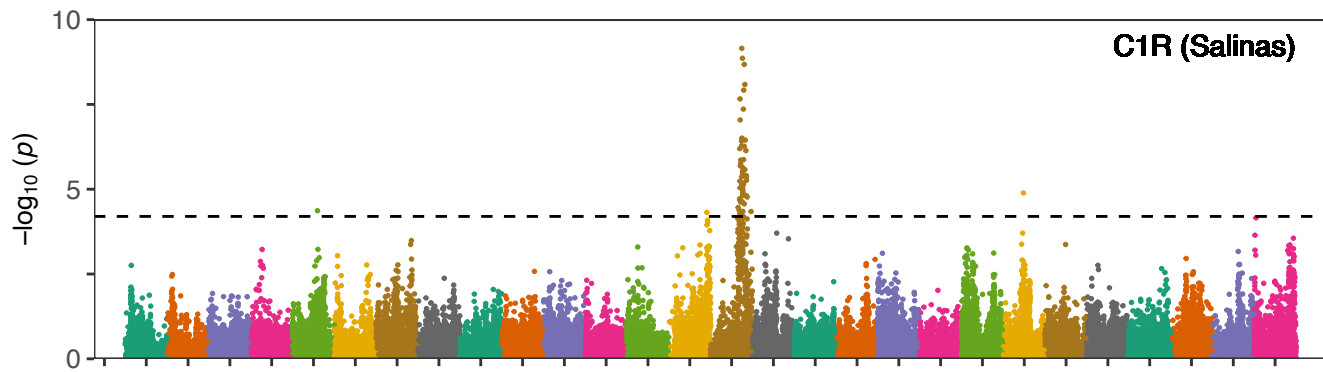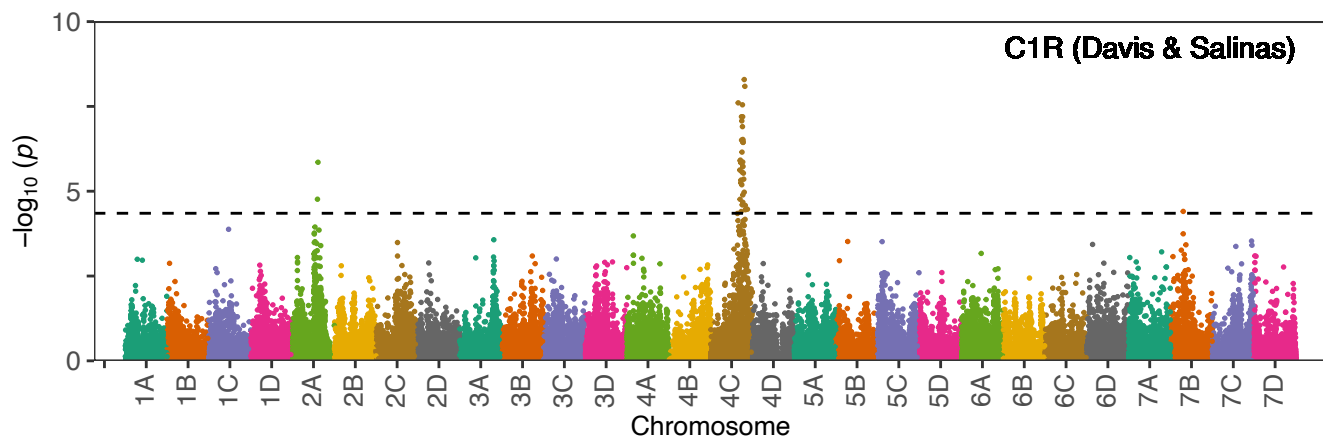

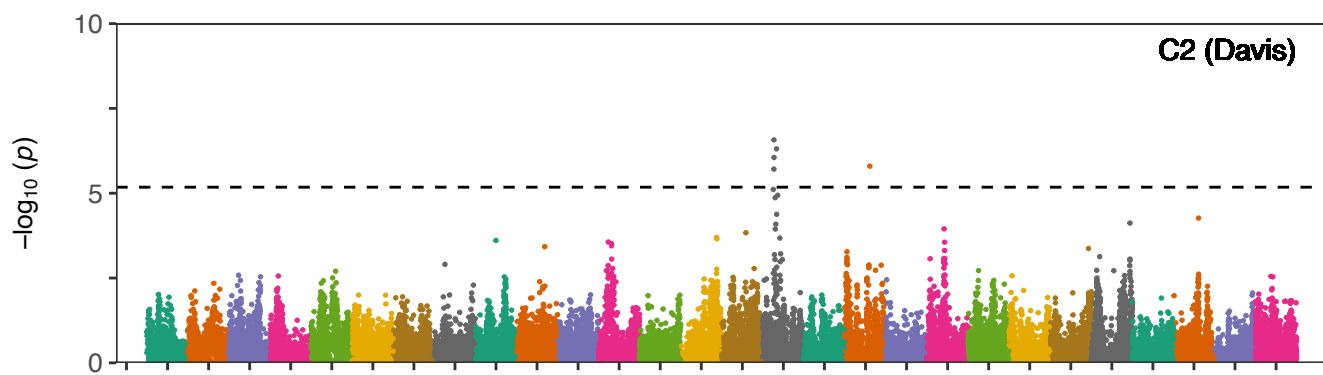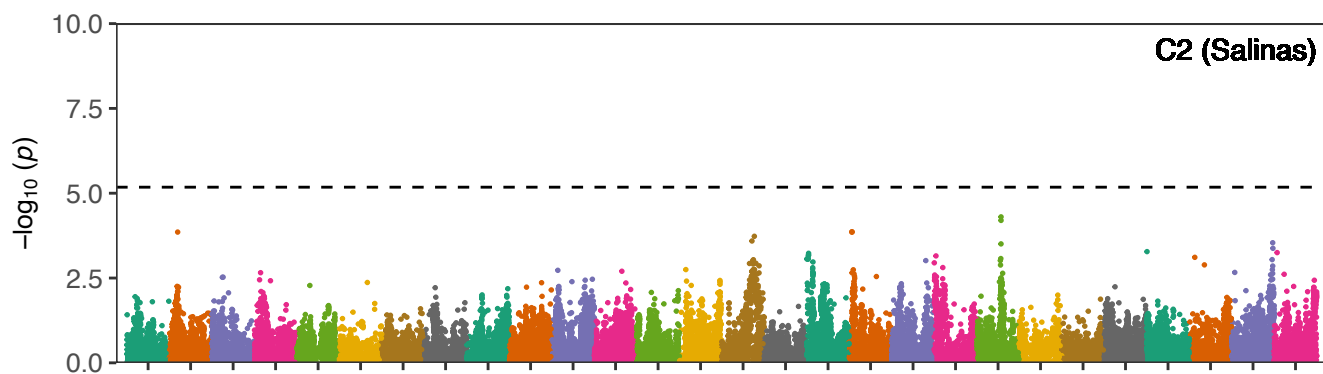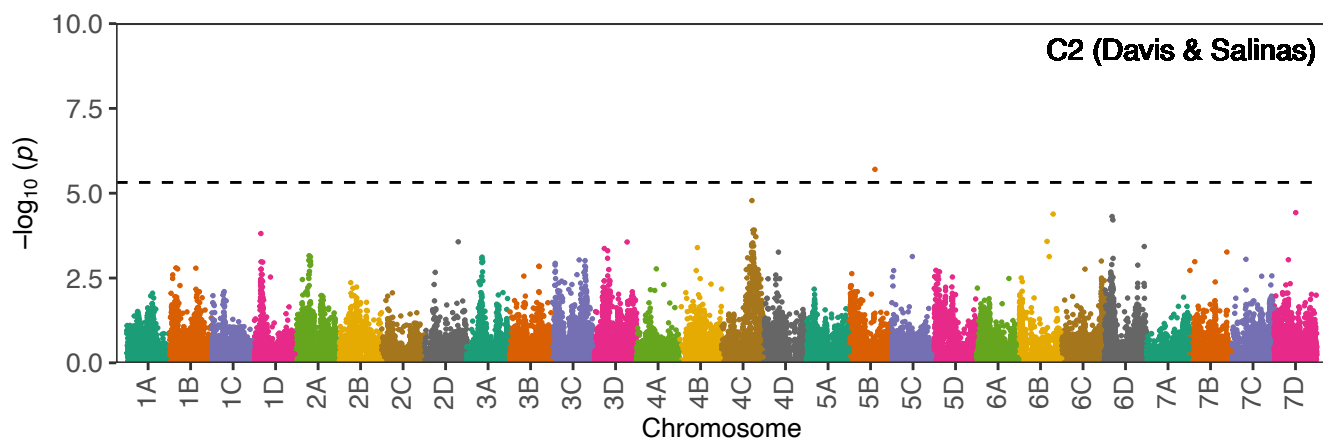

Supplement: Web_Material_uhad289 [file web_material_uhad289.zip › Supplemental Fig. S1 UC MANHATTAN 8-31-2023.pdf]

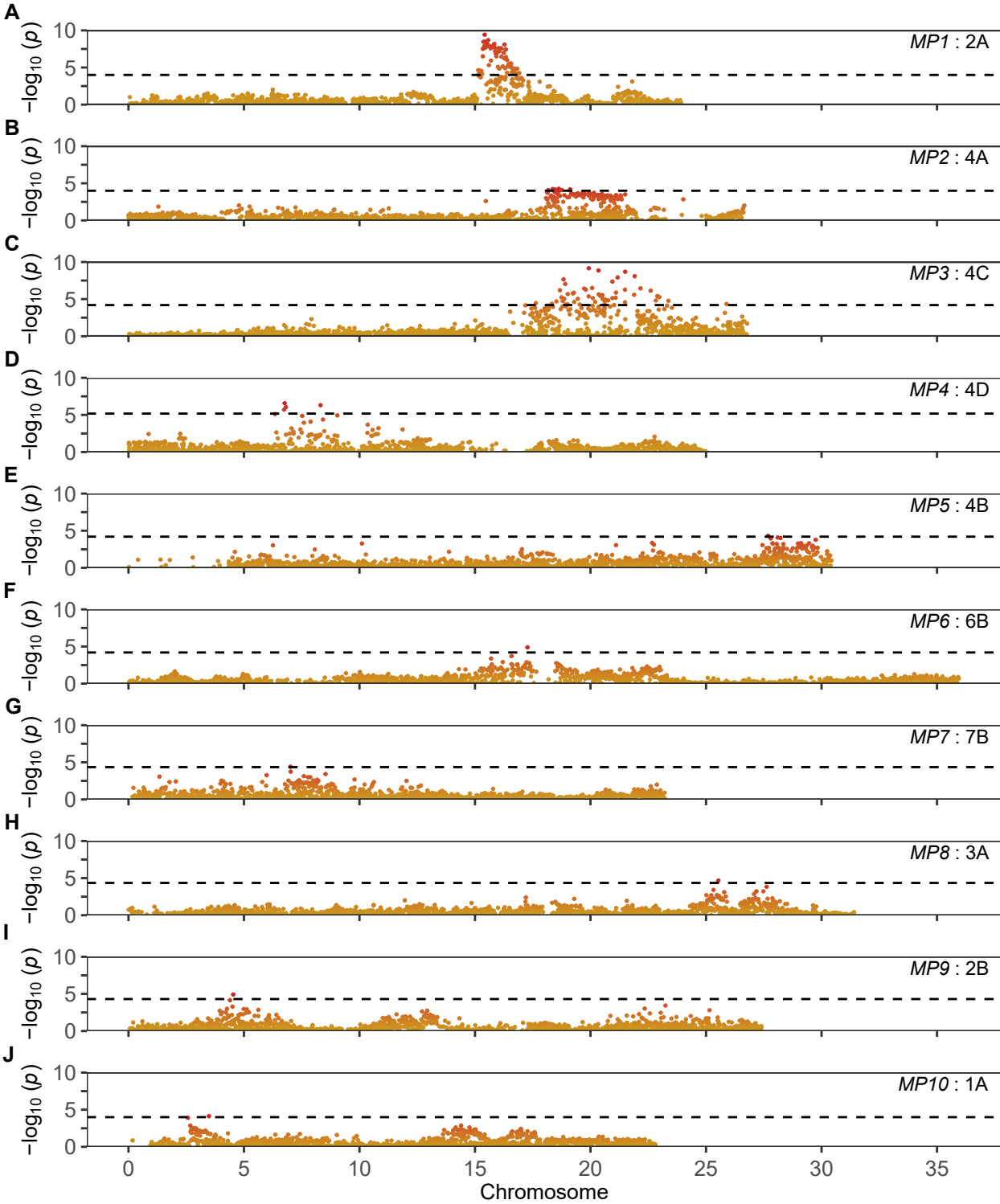

Supplement: Web_Material_uhad289 [file web_material_uhad289.zip › Supplemental Fig. S2 ZOOMED MANHATTAN 9-11-2023.pdf]

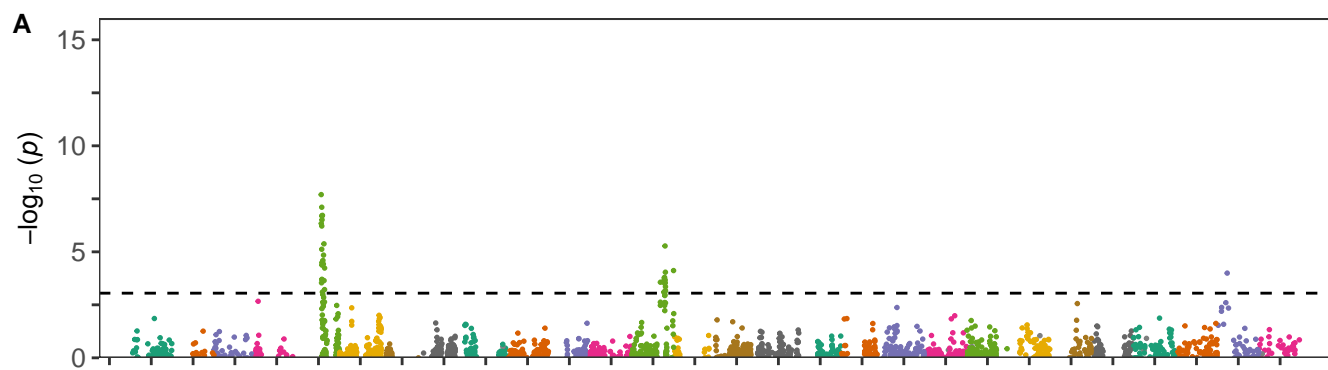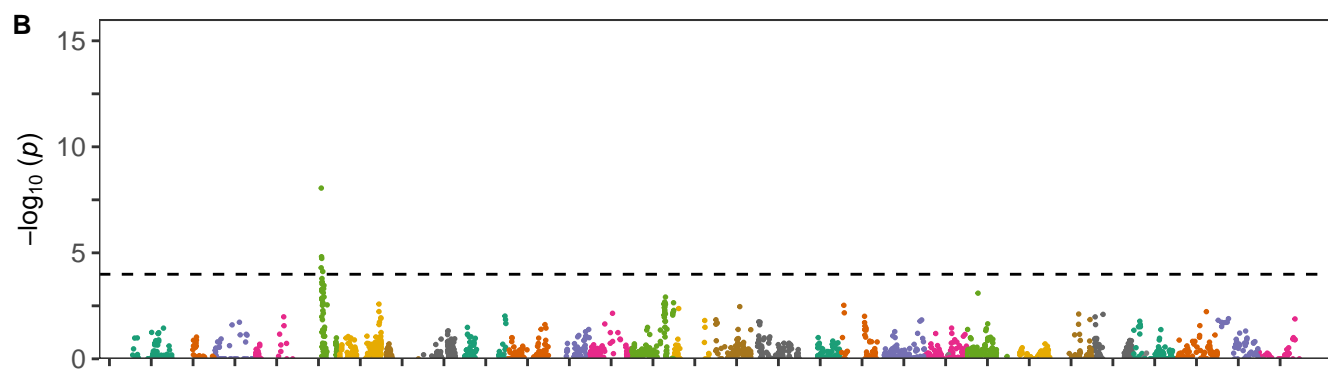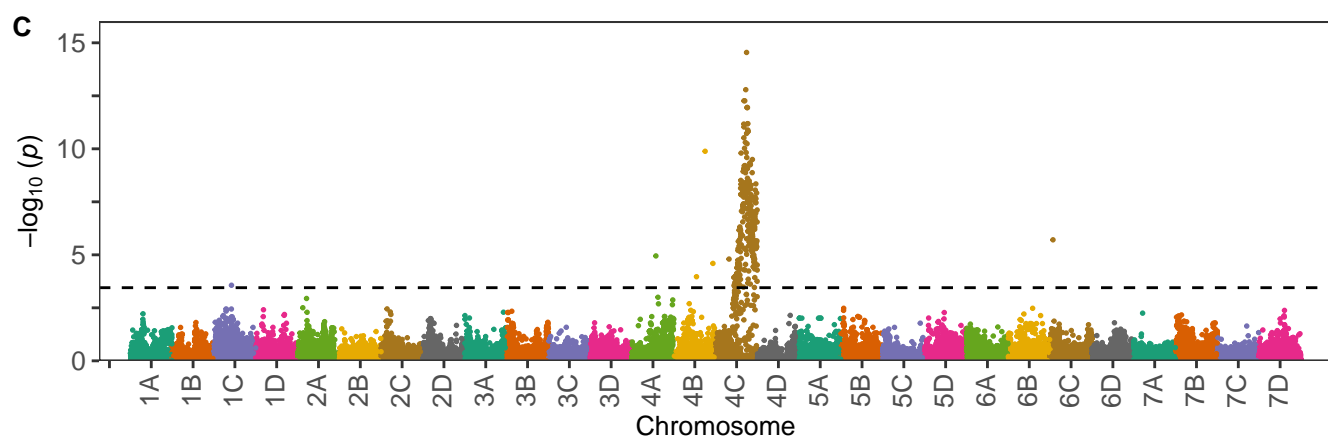

Supplement: Web_Material_uhad289 [file web_material_uhad289.zip › Supplemental Fig. S3 UF MANHATTAN 9-11-2023.pdf]
